# Supplementary material for: Clonal hematopoiesis is associated with adverse outcomes in multiple myeloma patients undergoing transplant
Source: Nat Commun. 2020 Jun 12;11:2996. doi: 10.1038/s41467-020-16805-5 (PMC7293239; doi:10.1038/s41467-020-16805-5)

Clonal hematopoiesis is associated with adverse outcomes in multiple myeloma patients undergoing transplant

Mouhieddine et al.

Supplementary Information

Table of Contents

Section		Page
Supplementary Table 1	List of genes in the targeted sequencing panel	3
Supplementary Table 2	List of queried genes and variants from the targeted sequencing data	4
Supplementary Table 3	Called somatic variants at ASCT	9
Supplementary Table 4	Distribution of mutated genes by age groups	12
Supplementary Table 5	Called somatic variants at TMN	13
Supplementary Table 6	Sequential samples of patients who developed a TMN following ASCT	14
Supplementary Table 7	Hematologic and immunologic parameters of patients at diagnosis	15
Supplementary Table 8	Hematologic and immunologic parameters of patients post induction	17
Supplementary Table 9	Absolute and percentage change in immunologic parameters of patients between diagnosis and post induction	19
Supplementary Figure 1	Study workflow	21
Supplementary Figure 2	Mutational properties	22
Supplementary Figure 3	Comparison of hematologic parameters of patients with CHIP vs. patients without CHIP at diagnosis	23
Supplementary Figure 4	Comparison of hematologic parameters of patients with CHIP vs. patients without CHIP post induction	24
Supplementary Figure 5	Effect of number of CHIP mutations on OS and PFS	25
Supplementary Figure 6	OS and PFS of patients divided into IMiD, non-IMiD and None	26
Supplementary Figure 7	Multivariable cox regression model of CHIP and IMiD interaction	27
Supplementary Figure 8	PFS and OS of patients with DNMT3A and TET2 mutations with respect to IMiD maintenance	28
Supplementary Figure 9	OS and PFS of patients with respect to the p.R882 DNMT3A mutation	29

SUPPLEMENTARY TABLES

Supplementary Table 1. List of genes in the targeted sequencing panel.

ABL1	CEBPA	FBXO36	MIR-142	PTPN11	STAT5B
ACTG1	CHD2	FBXW7	MOGAT3	PTPRF	STK11
AF088750	CKM	FGFR1	MPEG1	RAD21	SUZ12
AKT1	COASY	FGFR2	MPL	RASA2	TERC
ALK	CREBBP	FLT3	MRE11A	RASGRF1	TERT
ANGPTL7	CSF1R	FNDC3A	MTOR	RB1	TERT_COREPROMOTER
APC	CSF3R	FSIP2	MYCN	RET	TET2
ARHGAP26	CSNK1A1	GATA1	MYD88	RIT1	TGDS
ASXL1	CTCF	GATA2	MYH9	RNF151	TMA16
ASXL2	CTNNB1	GCSAM	NDUFAF3	ROBO1	TNFAIP3
ATM	CUX1	GNAS	NECAB3	RP11	TNFRSF14
ATR	CXCR4	GNB1	NF1	RPL10	TNKS
ATRX	CYLD	GRIA2	NFKBIA	RPL11	TP53
B2M	DCLK1	HIST1H1E	NOL4L	RPS7	TRAF3
BC062305	DDX41	HIST1H3G	NOTCH1	RUNX1	TRIP12
BCCIP	DENND4A	HIST1H3H	NOTCH2	RYR2	U2AF1
BCL2	DHX32	HNRNPK	NPM1	SETBP1	U2AF2
BCL7A	DIS3	HRAS	NRAS	SETD2	ULK4
BCOR	DNASE1L1	IDH1	NXF1	SF1	USP50
BCORL1	DNMT3A	IDH2	OR1L8	SF3A1	USP7
BIRC3	EED	IKZF1	OR1N2	SF3B1	VHL
BRAF	EGFR	INIP	OR1S2	SH2B3	VPS45
BRCC3	EGR1	IRF4	PDGFRA	SI	WHSC1
C15orf59	EIF4G2	JAK2	PDS5B	SLC24A1	WT1
C17orf77	ELAC1	JAK3	PHF6	SMAD4	XPO1
C20orf144	ENST00000520401	KCNMB3	PHIP	SMARCA4	YLPM1
CALR	EP300	KDM6A	PHOX2B	SMARCB1	YTHDF2
CARD11	ERBB2	KIT	PIGA	SMC1A	ZBTB33
CBL	ERBB4	KMT2D	PIGT	SMC3	ZFHX3
CBLB	ETNK1	KRAS	PIK3CA	SNX7	ZFHX4
CCDC144NL	ETV6	KRTDAP	PLA2G2D	SP140	ZMYM3
CCND1	EXOG	LPAR6	PNRC1	SP140L	ZNF318
CD58	EZH2	LRRC16A	PPM1D	SRCAP	ZP4
CDH1	FAM153A	LRRK2	PRDM1	SRP19	ZRSR2
CDK4	FAM153B	LTB	PRIM2	SRSF2_HOTSPOT	
CDKN1A	FAM46C	LUC7L2	PRPF40B	STAG1	
CDKN1B	FAM5C	MAX	PRPF8	STAG2	
CDKN2A	FANCL	MET	PTEN	STAT3	

Supplementary Table 2. List of queried genes and variants from the targeted sequencing data.

Gene Name	Mutations used for variant calling
ASXL1	Frameshift/nonsense/splice-site in exon 11-12; aa_range: 400-1540
ASXL2	Frameshift/nonsense/splice-site in exon 11-12
ATM	Frameshift/nonsense/splice-site
ATRX	Frameshift/nonsense/splice-site
B2M	Frameshift/nonsense/splice-site
BCOR	Frameshift/nonsense/splice-site
BCORL1	Frameshift/nonsense/splice-site
BIRC3	Frameshift/nonsense/splice-site in exon 2
BRAF	G464E, G464V, G466E, G466V, G469R, G469E, G469A, G469V, V471F, V472S, L485W, N581S, I582M, I592M, I592V, D594N, D594G, D594V, D594E, F595L, F595S, G596R, L597V, L597S, L597Q, L597R, A598V, V600M, V600L, V600K, V600R, V600E, V600A, V600G, V600D, K601E, K601N, R603*, W604R, W604G, S605G, S605F, S605N, G606E, G606A, G606V, H608R, H608L, G615R, S616P, S616F, L618S, L618W
BRCC3	Frameshift/nonsense/splice-site
CALR	Frameshift and aa_range: 352-418
CARD11	E93D, G123S, G126D, T128M, F130I, R179W, K182N, M183L, K215M, D230N, L232LI, M240MGLNKM, K244T, S250P, S250P, L251P, L251P, V266*, D338G, T353*, D357V, Y361H, M365K, D387V, D387V, D401V, R418S, R423W, E432K, E626K
CBL	Missense/frameshift/non-frameshift p.360-430 OR splice site
CBLB	Missense p.360-430
CDKN2A	Frameshift/nonsense/splice-site
CEBPA	Frameshift/nonsense/splice-site and missense and ExAc<0.001
CHD2	H620L, F1146L, L1270F
CREBBP	Frameshift/nonsense/splice-site, D1435E, R1446L, R1446H, R1446C, Y1450C, P1476R, Y1482H, H1487Y, W1502C, Y1503D, Y1503H, Y1503F, S1680del
CSF1R	Frameshift/nonsense, L301F, L301S, Y969C, Y969N, Y969F, Y969H, Y969D
CSF3R	Frameshift/nonsense p.618-840 (truncating c.741-791), T615A, T618I
CSNK1A1	E98, D140, missense with ExAC frequency <0.0001
CTC1	Frameshift/nonsense/splice-site
CTCF	Frameshift/nonsense, R377C, R377H, P378A, P378L
CUX1	Frameshift/nonsense/splice-site
DDX41	Frameshift/nonsense/splice-site, missense and aa_range: 360-430 with ExAC_Freq<0.0001

DNMT3A	Frameshift/nonsense/splice-site, F290I, F290C, V296M, P307S, P307R, R326H, R326L, R326C, R326S, G332R, G332E, V339A, V339M, V339G, L344Q, L344P, R366P, R366H, R366G, A368T, A368V, R379H, R379C, I407T, I407N, I407S, F414L, F414S, F414C, A462V, K468R, C497G, C497Y, Q527H, Q527P, Y533C, S535F, C537G, C537R, G543A, G543S, G543C, L547H, L547P, L547F, M548I, M548K, G550R, W581R, W581G, W581C, R604Q, R604W, R635W, R635Q, S638F, G646V, G646E, L653W, L653F, I655N, V657A, V657M, R659H, Y660C, V665G, V665L, M674V, R676W, R676Q, G685R, G685E, G685A, D686Y, D686G, R688H, G699R, G699S, G699D, P700L, P700S, P700R, P700Q, P700T, P700A, D702N, D702Y, V704M, V704G, I705F, I705T, I705S, I705N, G707D, G707V, C710S, C710Y, S714C, V716D, V716F, V716I, N717S, N717I, P718L, R720H, R720G, K721R, K721T, Y724C, R729Q, R729W, R729G, F731C, F731L, F731Y, F731I, F732del, F732C, F732S, F732L, E733G, E733A, F734L, F734C, Y735C, Y735N, Y735S, R736H, R736C, R736P, L737H, L737V, L737F, L737R, A741V, P742P, P743R, P743L, R749C, R749L, R749H, R749G, F751L, F751C, F752del, F752C, F752L, F752I, F752V, W753G, W753C, W753R, L754P, L754R, L754H, F755S, F755I, F755L, M761I, M761V, G762C, V763I, S770L, S770W, S770P, R771Q, F772I, F772V, L773R, L773V, E774K, E774D, E774G, I780T, D781G, R792H, W795C, W795L, G796D, G796V, N797Y, N797H, N797S, P799S, P799R, P799H, R803S, R803W, P804L, P804S, K826R, S828N, K829R, T835M, N838D, K841Q, Q842E, P849L, D857N, W860R, E863D, F868S, G869S, G869V, M880V, S881R, S881I, R882H, R882P, R882C, R882G, A884P, A884V, Q886R, L889P, L889R, G890D, G890R, G890S, V895M, P896L, V897G, V897D, R899L, R899H, R899C, L901R, L901H, P904L, F909C, P904Q, A910P, C911R, C911Y
EED	Frameshift/nonsense/splice-site, L240Q, I363M
EP300	Frameshift/nonsense/splice-site, VF1148_1149del, D1399N, D1399Y, P1452L, Y1467N, Y1467H, Y1467C, R1627W, A1629V
ETNK1	N244S, N244T, N244K
ETV6	Frameshift/nonsense/splice-site, missense and aa_range: 338-424 and #ETS domain and ExAC_Freq<0.0001, missense and aa_range: 56-123 and #PNT domain and ExAC_Freq<0.0001
EZH2	Frameshift/nonsense/splice-site, Q62R, N102S, F145S, F145C, F145Y, F145L, G159R, E164D, R202Q, K238E, E244K, R283Q, H292R, P488S, R497Q, R561H, T568I, K629E, Y641N, Y641H, Y641S, Y641C, Y641F, D659Y, D659G, V674M, A677G, A677V, R679C, R679H, R685C, R685H, A687V, N688I, N688K, H689Y, S690P, I708V, I708T, I708M, E720K, E740K
FAM46C	Frameshift/nonsense/splice-site
FANCL	Frameshift/nonsense/splice-site
FLT3	frameshift and aa_range: 569-648, nonframeshift and aa_range: 569-700, nonframeshift and aa_range: 800-850, V579A, V592A, V592I, F594L, FY590-591GD, N676, N663, F691, D835Y, D835H, D835E, del835, N841, Y842
GATA1	Frameshift/nonsense/splice-site, missense and ExAC_Freq<0.0001
GATA2	Frameshift/nonsense/splice-site, R293Q, N317H, A318T, A318V, A318G, G320D, L321P, L321F, L321V, Q328P, R330Q, R361L, L359V, A372T, R384G, R384K, position:128202118,128202197
GATA3	Frameshift/nonsense/splice-site ZNF domain, R276W, R276Q, N286T, L348V
GNA13	I34T, G57S, S62F, M68K, Q134R, Y145F, L152F, E167D, Q169H, R264H, E273K, V322G, V362G, L371F
GNAS	R201(844)S, R201(844)C, R201(844)H, R201(844)L, Q227(870)K, Q227(870)R, Q227(870)L, Q227(870)H, R374(1017)C
GNB1	K57N, K57M, K57E, K57T, D76, K78, I80T, I80N, K89, N88

HIST1H1E	A158T, A167V, P196S, K202E, K205R
IDH1	Nonframeshift and aa_range: 126-138 , R132C, R132G, R132H, R132L, R132P, R132V, V178I
IDH2	Nonframeshift and aa_range: 134-146, nonframeshift and aa_range: 164-180, R140W, R140Q, R140L, R140G, R172W, R172G, R172K, R172T, R172M, R172N, R172S
IKZF1	Frameshift/nonsense/splice-site
IKZF2	Frameshift/nonsense
IKZF3	Frameshift/nonsense
IRF4	N2S, S18T, I32V, L40V, Q60K, Q60H
JAK1	T478A, T478S, V623A, A634D, L653F, R724H, R724Q, R724P, T782M, L783F, N533D, N533Y, N533S, H538R, K539E, K539L, I540T, I540V, V617F, R683S, R683G, V683, R867, D873, P933, del/ins537-539L, del/ins538-539L, del/ins540-543MK, del/ins540-544MK, del/ins541-543K, del542-543, del543-544, ins11546-547
JAK3	M511T, M511I, A572V, A572T, A573V, R657Q, V715I, V715A
KDM6A	Frameshift/nonsense/splice-site, del419
KIT	frameshift, ins503, V559A, V559D, V559G, V559I, V560D, V560A, V560G, V560E, del560, E561K, del579, P627L, P627T, R634W, K642E, K642Q, V654A, V654E, H697Y, H697D, E761D, K807R, D816H, D816Y, D816F, D816I, D816V, D816H, del551-559
KRAS	G12D, G12A, G12E, G12V, G13D, G13C, G13Y, G13F, G13R, G13A, G13V, G13E, V14I, T58I, G60D, G60A, G60V, Q61K, Q61E, Q61P, Q61R, Q61L, Q61H, K117E, K117N, A146T, A146P, A146V
LRRK2	E155K, I543S
LTB	Frameshift/nonsense
LUC7L2	Frameshift/nonsense/splice-site
MIR142	ExAC_Freq<0.0001
MLL	Frameshift/nonsense
MLL2	Frameshift/nonsense
MPL	S505G, S505N, S505C, L510P, del513, W515A, W515R, W515K, W515S, W515L, A519T, A519V, Y591D, W515-518KT
MRE11A	Frameshift/nonsense/splice-site
MYD88	L265, V217F , S219C, M240T , S251N , P266 , L273P
NF1	Frameshift/nonsense/splice-site
NOTCH1	Missense and aa_range: 617-738 andExAC_Freq<0.0001, missense and aa_range: 500-616 andExAC_Freq<0.0001, frameshift/nonsense and aa_range: 2061-2555
NOTCH2	Frameshift/nonsense and aa_range: 2010-2471
NPM1	Frameshift p.W288fs (insertion at c.859_860, 860_861, 862_863, 863_864))
NRAS	G12S, G12R, G12C, G12N, G12P, G12Y, G12D, G12A, G12V, G12E, G13S, G13R, G13C, G13N, G13P, G13Y, G13D, G13A, G13V, G13E, G60E, G60R, Q61R, Q61L, Q61K, Q61P, Q61H, Q61Q, T74P, A146P
PDS5B	Frameshift/nonsense/splice-site, R1292Q
PDSS2	Frameshift/nonsense
PHF6	Frameshift/nonsense/splice-site, A40D, M125I, S246Y, F263L, R274Q, C297Y, H302Y, H329L
PHIP	Frameshift/nonsense/splice-site

PIGA	Frameshift/nonsense, missense and ExAC_Freq<0.0001
PIGT	Frameshift/nonsense
PPM1D	Frameshift/nonsense, exon 5 or 6
PRPF40B	Frameshift/nonsense/splice-site, P15H, M58I, P405L, P562S
PRPF8	Frameshift/nonsense/splice-site, M1307I, C1594W, D1598Y, D1598N, D1598V
PTEN	Frameshift/nonsense/splice-site, D24G, R47G, F56V, L57W, H61R, K66N, Y68H, C71Y, F81C, Y88C, D92G, D92V, D92E, H93Y, H93D, H93Q, N94I, P95L, I101T, C105F, C105S, D107Y, L112V, H123Y, C124R, C124S, K125E, A126D, K128N, R130G, R130Q, R130L, G132D, I135V, I135K, C136R, C136F, K144Q, A151T, D153Y, D153N, Y155H, Y155C, R159K, R159S, R161K, R161I, G165R, G165E, S170N, S170I, R173C, Y174D, Y177C, H196Y, R234W, G251C, D252Y, F271S, D326G
PTEN	Missense and ExAC_Freq<0.0001
PTPN11	G60V, G60R, G60A, D61Y, D61V, D61G, Y63C, E69K, E69G, E69D, E69Q, F71L, F71K, A72T, A72V, A72D, T73I, E76K, E76Q, E76M, E76A, E76G, E139G, E139D, N308D, N308T, N339S, P491L, S502P, S502A, S502L, G503V, G503G, G503A, G503E, Q506P, T507A, T507K, missense and ExAC_Freq<0.0001
RAD21	Frameshift/nonsense/splice-site, R65Q, H208R, Q474R
RIT1	S35, A57, F82, G95, A77, E81, T83, Y89, M90
RPL11	Frameshift/nonsense, missense and ExAC_Freq<0.0001
RPS7	Frameshift/nonsense, missense and ExAC_Freq<0.0001
RUNX1	Frameshift/nonsense/splice-site, S73F, H78Q, H78L, R80C, R80P, R80H, L85Q, P86L, P86H, S114L, D133Y, L134P, R135G, R135K, R135S, R139Q, R142S, A165V, R174Q, R177L, R177Q, A224T, D171G, D171V, D171N, R205W, R223C, missense and ExAC_Freq<0.0001
SETBP1	D868N, D868T, S869N, G870S, I871T, D880N, D880Q
SETD2	Frameshift/nonsense/splice-site, V1190M
SETDB1	Frameshift/nonsense, K715E
SF1	Frameshift/nonsense/splice-site, T454M, Y476C, A508G
SF3A1	Frameshift/nonsense/splice-site, A57S, M117I, K166T, Y271C, G347V, R387W, R387Q, E592K, E622D, Y623C, R625L, R625C, R625G, H662Q, H662D, T663I, K666N, K666T, K666E, K666R, K700E, V701F, A708T, G740R, G740E, A744P, D781G, E783K, R831Q, L833F, E862K, R957Q
SFRS2	Y44H, P95H, P95L, P95T, P95R, P95A, P107H, P95fs
SH2B3	Frameshift/nonsense, missense and aa_range: 195-307, missense and aa_range: 364-441
SMC1A	Frameshift/nonsense/splice-site, K190T, R586W, M689V, R807H, R1090H, R1090C
SMC3	Frameshift/nonsense/splice-site, R155I, Q367E, D392V, K571R, R661P, G662C
SRSF2	Frameshift and aa_range: 85-100, nonframeshift and aa_range: 85-100, P95
STAG1	Frameshift/nonsense/splice-site, H1085Y
STAG2	Frameshift/nonsense/splice-site
STAT3	Missense/nonframeshift and aa_range:584-674
STAT5B	missense/nonframeshift and aa_range:593-670
SUZ12	Frameshift/nonsense
TERC	ExAC_Freq<0.0001
TERT	Frameshift/nonsense, missense and ExAC_Freq<0.0001, nonframeshift and ExAC_Freq<0.0001

TET2	Frameshift/nonsense/splice-site, missense/nonframeshift mutations in catalytic domains (p.1104-1481 and 1843-2002)
TNFAIP3	Frameshift/nonsense, D117V, M476I, P574I
TP53	Frameshift/nonsense/splice-site, S46F, G105C, G105R, G105D, G108S, G108C, R110L, R110C, T118A, T118R, T118I, S127F, S127Y, L130V, L130F, K132Q, K132E, K132W, K132R, K132M, K132N, F134V. F134L, F134S, C135W, C135S, C135F, C135G, C135Y, Q136K, Q136E, Q136P, Q136R, Q136L, Q136H, A138P, A138V, A138A, A138T, T140I, C141R, C141G, C141A, C141Y, C141S, C141F, C141W, V143M, V143A, V143E, L145Q, W146C, W146L, L145R, V147G, P151T, P151A, P151S, P151H, P151R, P152S, P152R, P152L, T155P, T155A, V157F, R158H, R158L, A159V, A159P, A159S, A159D, A161T, A161D, Y163N, Y163H, Y163D, Y163S, Y163C, K164E, K164M, K164N, K164P, H168Y, H168P, H168R, H168L, H168Q, M169I, M169T, M169V, E171K, E171Q, E171G, E171A, E171V, E171D, V172D, V173M, V173L, V173G, R174W, R175G, R175C, R175H, C176R, C176G, C176Y, C176F, C176S, P177R, P177R, P177L, H178D, H178P, H178Q, H179Y, H179R, H179Q, R181C, R181Y, D186G, G187S, P190L, P190T, H193N, H193P, H193L, H193R, L194F, L194R, I195F, I195N, I195Tl R196Pl V197L, G199V, Y205N, Y205C, Y205H, D208V, R213Q, R213P, R213L, R213Q, H214D, H214R, S215G, S215I, S215R, V216M, V217G, Y220N, Y220H, Y220S, Y220C, E224D, I232F, I232N, I232T, I232S, Y234N, Y234H, Y234S, Y234C, Y236N, Y236H, Y236C, M237V, M237K, M237I, C238R, C238G, C238Y, C238W, N239T, N239S, S241Y, S241C, S241F, C242G, C242Y, C242S, C242F, G244S, G244C, G244D, G245S, G245R, G245C, G245D, G245A, G245V, G245S, M246V, M246K, M246R, M246I, N247I, R248W, R248G, R248Q, R249G, R249W, R249T, R249M, P250L, I251N, L252P, I254S, I255F, I255N, I255S, L257Q, L257P, E258K, E258Q, D259Y, S261T, G262D, G262V, L265P, G266R, G266E, G266V, R267W, R267Q, R267P, E271K, V272M, V272L, R273S, R273G, R273C, R273H, R273P, R273L, V274F, V274D, V274A, V274G, V274L, C275Y, C275S, C275F, A276P, C277F, C277Y, P278T, P278A, P278S, P278H, P278R, P278L, G279E, R280G, R280K, R280T, R280I, R280S, D281N, D281H, D281Y, D281G, D281E, R282G, R282W, R282Q, R282P, E285K, E285V, E286G, E286V, E286K, K320N, L330R, G334V, R337C, R337L, A347T, L348F, T377P, missense and ExAC_Freq<0.0001
TRAF3	Frameshift/nonsense
U2AF1	Nonframeshift and aa_range: 30-38, nonframeshift and aa_range: 153-161, D14G, S34F, S34Y, R35L, R156H, R156Q, Q157R, Q157P
U2AF2	R18W, Q143L, M144I, L187V, Q190L, nonframeshift and ExAC_Freq<0.0001
VPS45	Frameshift/nonsense, missense/nonframeshift and ExAC_Freq<0.0001
WT1	Frameshift/nonsense/splice-site
XPO1	E571A, E571K
ZRSR2	Frameshift/nonsense/splice-site, R126P, E133G, C181F, H191Y, I202N, F239V, F239Y, N261Y, C280R, C302R, C326R, H330R, N382K

Supplementary Table 3. Called somatic variants at ASCT.

Gene	Chromosome	Start Position	End Position	Variant Classification	Variant Type	Reference Allele	Tumor_Seq_Allele1	Tumor_Seq_Allele2	Codon_Change	cDNA_Change	Protein Change	# Reference Reads	# Alternate Reads	Total Depth	TLOD Score	QSI_NT Score	Variant Allele Frequency	Sample
ASXL1	20	31021535	31021535	Nonsense_Mutation	SNP	C	C	T	c.(1534-1536)Cag>Tag	c.1534C>T	p.Q512*	534	412	946	1179.90805	NA	0.435518	CHIP562
ASXL1	20	31022441	31022442	Frame_Shift_Ins	INS	-	-	G	c.(1927-1929)gggfs	c.1926_1927insG	p.G643fs	616	230	846	NA	332	0.27186761	CHIP418
ASXL1	20	31022441	31022442	Frame_Shift_Ins	INS	-	-	G	c.(1927-1929)gggfs	c.1926_1927insG	p.G643fs	379	135	514	NA	304	0.26264591	CHIP374
ASXL1	20	31021185	31021186	Frame_Shift_Ins	INS	-	-	A	c.(1183-1188)atacagfs	c.1184_1185insA	p.Q396fs	1241	191	1432	NA	427	0.13337989	CHIP189
ASXL1	20	31024279	31024279	Nonsense_Mutation	SNP	C	C	G	c.(3763-3765)tCa>tGa	c.3764C>G	p.S1255*	807	119	926	303.499177	NA	0.12851	CHIP277
ASXL1	20	31023087	31023087	Nonsense_Mutation	SNP	C	C	T	c.(2572-2574)Cag>Tag	c.2572C>T	p.Q858*	903	42	945	84.687015	NA	0.044444	CHIP36
ASXL1	20	31023411	31023412	Frame_Shift_Ins	INS	-	-	GAGGCAGTGA	c.(2896-2898)ggafs	c.2896_2897insGAGGCAGTGA	p.-966fs	1562	67	1629	NA	399	0.04112953	CHIP589
ASXL1	20	31024758	31024758	Nonsense_Mutation	SNP	C	C	T	c.(4243-4245)Cga>Tga	c.4243C>T	p.R1415*	869	28	897	52.635493	NA	0.031215	CHIP597
ASXL1	20	31021280	31021280	Nonsense_Mutation	SNP	A	A	T	c.(1279-1281)Aaa>Taa	c.1279A>T	p.K427*	929	23	952	40.455746	NA	0.02416	CHIP615
ASXL1	20	31021250	31021250	Nonsense_Mutation	SNP	C	C	T	c.(1249-1251)Cga>Tga	c.1249C>T	p.R417*	904	21	925	35.461165	NA	0.022703	CHIP204
ASXL1	20	31022902	31022902	Nonsense_Mutation	SNP	G	G	A	c.(2386-2388)tGg>tAg	c.2387G>A	p.W796*	835	12	847	19.532654	NA	0.014168	CHIP40
ASXL1	20	31022277	31022277	Nonsense_Mutation	SNP	C	C	T	c.(1762-1764)Cag>Tag	c.1762C>T	p.Q588*	876	12	888	12.453337	NA	0.013514	CHIP500
ASXL1	20	31024208	31024209	Frame_Shift_Ins	INS	-	-	A	c.(3694-3696)aaafs	c.3693_3694insA	p.K1232fs	1679	326	2005	NA	400	0.16259352	CHIP547
ATM	11	108121428	108121428	Splice_Site	SNP	G	G	T	c.(1234-1236)tgG>tgT	c.1236G>T	p.W412C	238	9	247	8.410378	NA	0.036437	CHIP284
ATM	11	108224493	108224493	Splice_Site	SNP	G	G	A	c.(8671-8673)gGt>gAt	c.8672G>A	p.G2891D	886	13	899	19.664766	NA	0.014461	CHIP252
ATM	11	108224493	108224493	Splice_Site	SNP	G	G	A	c.(8671-8673)gGt>gAt	c.8672G>A	p.G2891D	904	13	917	19.048341	NA	0.014177	CHIP160
BRCC3	X	154299826	154299835	Frame_Shift_Del	DEL	GGTGCAGGCG	GGTGCAGGCG	-	c.(22-33)gcggtgcaggcgfs	c.24_33delGGTGCAGGCG	p.AVQA8fs	622	62	684	NA	114	0.09064327	CHIP274
BRCC3	X	154306935	154306935	Nonsense_Mutation	SNP	G	G	A	c.(361-363)tgG>tgA	c.363G>A	p.W121*	537	29	566	61.685689	NA	0.051237	CHIP392
BRCC3	X	154299830	154299830	Nonsense_Mutation	SNP	C	C	T	c.(28-30)Cag>Tag	c.28C>T	p.Q10*	418	7	425	9.95882	NA	0.016471	CHIP131
CBL	11	119148931	119148931	Missense_Mutation	SNP	G	G	A	c.(1150-1152)tGt>tAt	c.1151G>A	p.C384Y	811	36	847	70.983522	NA	0.042503	CHIP123
CBL	11	119149250	119149250	Missense_Mutation	SNP	C	C	G	c.(1258-1260)Cga>Gga	c.1258C>G	p.R420G	914	21	935	34.961535	NA	0.02246	CHIP334
CBL	11	119148991	119148991	Missense_Mutation	SNP	G	G	A	c.(1210-1212)tGt>tAt	c.1211G>A	p.C404Y	893	9	902	12.022914	NA	0.009978	CHIP78
DNMT3A	2	25463212	25463213	Frame_Shift_Ins	INS	-	-	G	c.(2278-2283)gccatgfs	c.2280_2281insC	p.M761fs	449	243	692	NA	308	0.35115607	CHIP15
DNMT3A	2	25457243	25457243	Missense_Mutation	SNP	G	G	A	c.(2644-2646)Cgc>Tgc	c.2644C>T	p.R882C	349	177	526	540.008929	NA	0.336502	CHIP315
DNMT3A	2	25463182	25463182	Nonsense_Mutation	SNP	G	G	A	c.(2311-2313)Cga>Tga	c.2311C>T	p.R771*	462	118	580	319.573327	NA	0.203448	CHIP313
DNMT3A	2	25470516	25470516	Nonsense_Mutation	SNP	G	G	A	c.(958-960)Cga>Tga	c.958C>T	p.R320*	749	175	924	472.294106	NA	0.189394	CHIP225
DNMT3A	2	25457192	25457192	Missense_Mutation	SNP	G	G	A	c.(2695-2697)Cgc>Tgc	c.2695C>T	p.R899C	521	101	622	285.10352	NA	0.162379	CHIP355
DNMT3A	2	25457242	25457242	Missense_Mutation	SNP	C	C	T	c.(2644-2646)cGc>cAc	c.2645G>A	p.R882H	759	144	903	388.075759	NA	0.159468	CHIP489
DNMT3A	2	25457243	25457243	Missense_Mutation	SNP	G	G	A	c.(2644-2646)Cgc>Tgc	c.2644C>T	p.R882C	677	105	782	261.973206	NA	0.134271	CHIP243
DNMT3A	2	25467449	25467449	Missense_Mutation	SNP	C	C	A	c.(1627-1629)Ggc>Tgc	c.1627G>T	p.G543C	803	123	926	295.841789	NA	0.132829	CHIP313
DNMT3A	2	25467142	25467142	Missense_Mutation	SNP	T	T	C	c.(1732-1734)gAa>gGa	c.1733A>G	p.E578G	818	106	924	260.990479	NA	0.114719	CHIP536
DNMT3A	2	25457266	25457275	Frame_Shift_Del	DEL	TAGTGGACTG	TAGTGGACTG	-	c.(2611-2622)ccagtccactatfs	c.2612_2621delCAGTCCACTA	p.PVHY871fs	611	71	682	NA	223	0.10410557	CHIP621
DNMT3A	2	25457243	25457243	Missense_Mutation	SNP	G	G	A	c.(2644-2646)Cgc>Tgc	c.2644C>T	p.R882C	673	70	743	167.609384	NA	0.094213	CHIP502
DNMT3A	2	25470516	25470516	Nonsense_Mutation	SNP	G	G	A	c.(958-960)Cga>Tga	c.958C>T	p.R320*	812	84	896	192.567912	NA	0.09375	CHIP344
DNMT3A	2	25470557	25470557	Nonsense_Mutation	SNP	C	C	T	c.(916-918)tGg>tAg	c.917G>A	p.W306*	863	84	947	190.865778	NA	0.088701	CHIP430
DNMT3A	2	25457242	25457242	Missense_Mutation	SNP	C	C	T	c.(2644-2646)cGc>cAc	c.2645G>A	p.R882H	511	48	559	117.085073	NA	0.085868	CHIP21
DNMT3A	2	25457163	25457163	Nonsense_Mutation	SNP	A	A	C	c.(2722-2724)taT>taG	c.2724T>G	p.Y908*	781	73	854	183.440268	NA	0.08548	CHIP568
DNMT3A	2	25463234	25463234	Nonsense_Mutation	SNP	C	C	T	c.(2257-2259)tgG>tgA	c.2259G>A	p.W753*	546	48	594	105.996779	NA	0.080808	CHIP30
DNMT3A	2	25457235	25457242	Frame_Shift_Del	DEL	CGCCAAGC	CGCCAAGC	-	c.(2644-2652)cgcttggcgfs	c.2645_2652delGCTTGGCG	p.RLA882fs	859	74	933	NA	265	0.07931404	CHIP628
DNMT3A	2	25468888	25468888	Splice_Site	SNP	C	C	T	c.e12+1			849	67	916	145.394088	NA	0.073144	CHIP106
DNMT3A	2	25469065	25469072	Frame_Shift_Del	DEL	GCTTCTCC	GCTTCTCC	-	c.(1384-1395)gcggagaagcccfs	c.1386_1393delGGAGAAGC	p.EKP463fs	876	67	943	NA	371	0.07104984	CHIP598
DNMT3A	2	25467083	25467083	Nonsense_Mutation	SNP	G	G	A	c.(1792-1794)Cga>Tga	c.1792C>T	p.R598*	811	59	870	137.529586	NA	0.067816	CHIP594
DNMT3A	2	25464453	25464465	Frame_Shift_Del	DEL	ACGTCCCCGACGT	ACGTCCCCGACGT	-	c.(2047-2061)tacgtcggggacgtcfs	c.2048_2060delACGTCGGGGACGT	p.YVGDV683fs	1099	78	1177	NA	366	0.06627018	CHIP297
DNMT3A	2	25467023	25467023	Splice_Site	SNP	C	C	T	c.e15+1			887	59	946	125.365968	NA	0.062368	CHIP444
DNMT3A	2	25467029	25467029	Nonsense_Mutation	SNP	C	C	A	c.(1846-1848)Gaa>Taa	c.1846G>T	p.E616*	698	38	736	79.047726	NA	0.05163	CHIP377
DNMT3A	2	25468121	25468121	Splice_Site	SNP	C	C	T	c.e13+1			331	18	349	35.533302	NA	0.051576	CHIP599
DNMT3A	2	25469919	25469919	Splice_Site	SNP	C	C	T	c.e9+1			535	28	563	56.873835	NA	0.049734	CHIP373
DNMT3A	2	25457243	25457243	Missense_Mutation	SNP	G	G	T	c.(2644-2646)Cgc>Agc	c.2644C>A	p.R882S	643	31	674	63.944487	NA	0.045994	CHIP446
DNMT3A	2	25463320	25463320	Splice_Site	SNP	C	C	G	c.e19-1			381	16	397	34.631196	NA	0.040302	CHIP412
DNMT3A	2	25458695	25458695	Splice_Site	SNP	C	C	A	c.e22-1			848	32	880	63.597721	NA	0.036364	CHIP510
DNMT3A	2	25467110	25467110	Nonsense_Mutation	SNP	T	T	A	c.(1765-1767)Aag>Tag	c.1765A>T	p.K589*	822	28	850	45.725136	NA	0.032941	CHIP397
DNMT3A	2	25470494	25470494	Nonsense_Mutation	SNP	C	C	T	c.(979-981)tGg>tAg	c.980G>A	p.W327*	907	30	937	57.856872	NA	0.032017	CHIP573
DNMT3A	2	25467474	25467474	Missense_Mutation	SNP	C	C	A	c.(1600-1602)caG>caT	c.1602G>T	p.Q534H	888	29	917	49.202035	NA	0.031625	CHIP398
DNMT3A	2	25467466	25467466	Missense_Mutation	SNP	C	C	T	c.(1609-1611)tGc>tAc	c.1610G>A	p.C537Y	922	28	950	45.757013	NA	0.029474	CHIP601
DNMT3A	2	25468935	25468935	Splice_Site	SNP	T	T	A	c.e12-2			891	24	915	46.066155	NA	0.02623	CHIP490
DNMT3A	2	25463182	25463182	Nonsense_Mutation	SNP	G	G	A	c.(2311-2313)Cga>Tga	c.2311C>T	p.R771*	775	19	794	34.770541	NA	0.023929	CHIP537
DNMT3A	2	25457161	25457161	Missense_Mutation	SNP	A	A	G	c.(2725-2727)tTt>tCt	c.2726T>C	p.F909S	655	16	671	29.396107	NA	0.023845	CHIP373
DNMT3A	2	25470029	25470029	Splice_Site	SNP	T	T	C	c.e22-2			826	20	846	29.757536	NA	0.023641	CHIP510
DNMT3A	2	25467429	25467429	Missense_Mutation	SNP	G	G	C	c.(1645-1647)tgC>tgG	c.1647C>G	p.C549W	957	23	980	37.728595	NA	0.023469	CHIP431
DNMT3A	2	25461998	25461998	Splice_Site	SNP	C	C	T	c.e20+1			909	21	930	32.734001	NA	0.022581	CHIP547
DNMT3A	2	25467022	25467022	Splice_Site	SNP	A	A	T	c.e15+1			787	18	805	30.142126	NA	0.02236	CHIP356
DNMT3A	2	25466766	25466766	Splice_Site	SNP	C	C	T	c.e16+1			534	12	546	18.961188	NA	0.021978	CHIP510
DNMT3A	2	25471016	25471016	Nonsense_Mutation	SNP	G	G	A	c.(745-747)Cag>Tag	c.745C>T	p.Q249*	328	7	335	11.624455	NA	0.020896	CHIP628
DNMT3A	2	25457242	25457242	Missense_Mutation	SNP	C	C	T	c.(2644-2646)cGc>cAc	c.2645G>A	p.R882H	519	11	530	19.843215	NA	0.020755	CHIP81
DNMT3A	2	25467190	25467190	Missense_Mutation	SNP	C	C	T	c.(1684-1686)tGt>tAt	c.1685G>A	p.C562Y	847	16	863	26.998002	NA	0.01854	CHIP37
DNMT3A	2	25459876	25459876	Splice_Site	SNP	T	T	C	c.e21-2			328	6	334	10.23173	NA	0.017964	CHIP96
DNMT3A	2	25469028	25469028	Splice_Site	SNP	C	C	T	c.e11+1			887	16	903	24.248983	NA	0.017719	CHIP546
DNMT3A	2	25457161	25457161	Missense_Mutation	SNP	A	A	G	c.(2725-2727)tTt>tCt	c.2726T>C	p.F909S	858	15	873	25.104811	NA	0.017182	CHIP544
DNMT3A	2	25470535	25470535	Nonsense_Mutation	SNP	C	C	T	c.(937-939)tgG>tgA	c.939G>A	p.W313*	929	16	945	26.444573	NA	0.016931	CHIP296
DNMT3A	2	25457194	25457194	Missense_Mutation	SNP	A	A	G	c.(2692-2694)aTc>aCc	c.2693T>C	p.I898T	873	15	888	24.717427	NA	0.016892	CHIP498
DNMT3A	2	25462050	25462050	Nonsense_Mutation	SNP	G	G	C	c.(2356-2358)tCa>tGa	c.2357C>G	p.S786*	837	14	851	23.629452	NA	0.016451	CHIP154
DNMT3A	2	25469919	25469919	Splice_Site	SNP	C	C	T	c.e9+1			629	10	639	13.389552	NA	0.015649	CHIP510
DNMT3A	2	25463182	25463182	Nonsense_Mutation	SNP	G	G	A	c.(2311-2313)Cga>Tga	c.2311C>T	p.R771*	825	13	838	22.224375	NA	0.015513	CHIP165
DNMT3A	2	25467072	25467072	Nonsense_Mutation	SNP	C	C	T	c.(1801-1803)tgG>tgA	c.1803G>A	p.W601*	917	14	931	20.526374	NA	0.015038	CHIP152
DNMT3A	2	25457242	25457242	Missense_Mutation	SNP	C	C	T	c.(2644-2646)cGc>cAc	c.2645G>A	p.R882H	886	13	899	21.531619	NA	0.014461	CHIP4
DNMT3A	2	25457192	25457192	Missense_Mutation	SNP	G	G	A	c.(2695-2697)Cgc>Tgc	c.2695C>T	p.R899C	697	10	707	13.006995	NA	0.014144	CHIP540
DNMT3A	2	25458575	25458575	Splice_Site	SNP	C	C	T	c.e22+1			909	12	921	13.622799	NA	0.013029	CHIP271
DNMT3A	2	25467083	25467083	Nonsense_Mutation	SNP	G	G	A	c.(1792-1794)Cga>Tga	c.1792C>T	p.R598*	928	12	940	18.976424	NA	0.012766	CHIP583
DNMT3A	2	25461999	25461999	Splice_Site	SNP	C	C	A	c.(2407-2409)aGg>aTg	c.2408G>T	p.R803M	876	11	887	14.903052	NA	0.012401	CHIP489
DNMT3A	2	25457243	25457243	Missense_Mutation	SNP	G	G	A	c.(2644-2646)Cgc>Tgc	c.2644C>T	p.R882C	737	9	746	15.273848	NA	0.012064	CHIP363

DNMT3A	2	25467099	25467099	Nonsense_Mutation	SNP	G	G	C	c.(1774-1776)taC>taG	c.1776C>G	p.Y592*	926	11	937	15.540448	NA	0.01174	CHIP152
DNMT3A	2	25458595	25458595	Missense_Mutation	SNP	A	A	G	c.(2578-2580)Tgg>Cgg	c.2578T>C	p.W860R	807	9	816	12.655522	NA	0.011029	CHIP355
DNMT3A	2	25470620	25470620	Splice_Site	SNP	T	T	C	c.e8-2			938	10	948	11.052632	NA	0.010549	CHIP486
DNMT3A	2	25466768	25466768	Splice_Site	SNP	T	T	C	c.(1933-1935)acA>acG	c.1935A>G	p.T645T	668	7	675	8.693708	NA	0.01037	CHIP498
DNMT3A	2	25457243	25457243	Missense_Mutation	SNP	G	G	A	c.(2644-2646)Cgc>Tgc	c.2644C>T	p.R882C	882	9	891	14.101699	NA	0.010101	CHIP595
DNMT3A	2	25457243	25457243	Missense_Mutation	SNP	G	G	A	c.(2644-2646)Cgc>Tgc	c.2644C>T	p.R882C	892	9	901	11.107249	NA	0.009989	CHIP106
DNMT3A	2	25467158	25467158	Nonsense_Mutation	SNP	G	G	A	c.(1717-1719)Cag>Tag	c.1717C>T	p.Q573*	931	8	939	10.086567	NA	0.00852	CHIP489
DNMT3A	2	25457185	25457185	Missense_Mutation	SNP	A	A	T	c.(2701-2703)cTc>cAc	c.2702T>A	p.L901H	887	7	894	9.91702	NA	0.00783	CHIP322
DNMT3A	2	25467190	25467190	Missense_Mutation	SNP	C	C	T	c.(1684-1686)tGt>tAt	c.1685G>A	p.C562Y	906	6	912	7.529783	NA	0.006579	CHIP624
GNB1	1	1747229	1747229	Missense_Mutation	SNP	T	T	C	c.(169-171)Aag>Gag	c.169A>G	p.K57E	891	19	910	28.578741	NA	0.020879	CHIP198
GNB1	1	1747229	1747229	Missense_Mutation	SNP	T	T	C	c.(169-171)Aag>Gag	c.169A>G	p.K57E	846	13	859	19.913136	NA	0.015134	CHIP347
GNB1	1	1747229	1747229	Missense_Mutation	SNP	T	T	C	c.(169-171)Aag>Gag	c.169A>G	p.K57E	941	12	953	16.341784	NA	0.012592	CHIP444
GNB1	1	1747229	1747229	Missense_Mutation	SNP	T	T	C	c.(169-171)Aag>Gag	c.169A>G	p.K57E	927	10	937	12.921524	NA	0.010672	CHIP116
GNB1	1	1747229	1747229	Missense_Mutation	SNP	T	T	C	c.(169-171)Aag>Gag	c.169A>G	p.K57E	938	7	945	8.472628	NA	0.007407	CHIP394
JAK2	9	5073770	5073770	Missense_Mutation	SNP	G	G	T	c.(1849-1851)Gtc>Ttc	c.1849G>T	p.V617F	220	723	943	2332.02379	NA	0.766702	CHIP515
JAK2	9	5073770	5073770	Missense_Mutation	SNP	G	G	T	c.(1849-1851)Gtc>Ttc	c.1849G>T	p.V617F	663	99	762	209.275897	NA	0.129921	CHIP277
JAK2	9	5073770	5073770	Missense_Mutation	SNP	G	G	T	c.(1849-1851)Gtc>Ttc	c.1849G>T	p.V617F	791	64	855	124.62691	NA	0.074854	CHIP321
JAK2	9	5073770	5073770	Missense_Mutation	SNP	G	G	T	c.(1849-1851)Gtc>Ttc	c.1849G>T	p.V617F	836	14	850	15.870618	NA	0.016471	CHIP177
JAK3	19	17946791	17946791	Missense_Mutation	SNP	A	A	T	c.(1855-1857)gTg>gAg	c.1856T>A	p.V619E	351	6	357	7.419289	NA	0.016807	CHIP442
KDM6A	X	44913178	44913178	Nonsense_Mutation	SNP	C	C	T	c.(853-855)Cag>Tag	c.853C>T	p.Q285*	851	13	864	18.659236	NA	0.015046	CHIP45
KRAS	12	25398282	25398282	Missense_Mutation	SNP	C	C	A	c.(37-39)Ggc>Tgc	c.37G>T	p.G13C	846	72	918	155.623279	NA	0.078431	CHIP29
KRAS	12	25380276	25380276	Missense_Mutation	SNP	T	T	C	c.(181-183)cAa>cGa	c.182A>G	p.Q61R	855	47	902	84.479527	NA	0.052106	CHIP9
KRAS	12	25398285	25398285	Missense_Mutation	SNP	C	C	G	c.(34-36)Ggt>Cgt	c.34G>C	p.G12R	924	25	949	43.086678	NA	0.026344	CHIP273
KRAS	12	25398281	25398281	Missense_Mutation	SNP	C	C	T	c.(37-39)gGc>gAc	c.38G>A	p.G13D	826	21	847	37.260091	NA	0.024793	CHIP524
KRAS	12	25380276	25380276	Missense_Mutation	SNP	T	T	A	c.(181-183)cAa>cTa	c.182A>T	p.Q61L	907	12	919	17.56963	NA	0.013058	CHIP608
KRAS	12	25398281	25398281	Missense_Mutation	SNP	C	C	T	c.(37-39)gGc>gAc	c.38G>A	p.G13D	945	10	955	12.908763	NA	0.010471	CHIP630
NF1	17	29490204	29490204	Splice_Site	SNP	C	C	T	c.(289-291)Caa>Taa	c.289C>T	p.Q97*	766	13	779	22.038331	NA	0.016688	CHIP237
NF1	17	29528086	29528086	Nonsense_Mutation	SNP	C	C	G	c.(1093-1095)tCa>tGa	c.1094C>G	p.S365*	887	9	896	14.161076	NA	0.010045	CHIP631
PHIP	6	79664614	79664614	Nonsense_Mutation	SNP	G	G	A	c.(3970-3972)Cag>Tag	c.3970C>T	p.Q1324*	821	55	876	117.433646	NA	0.062785	CHIP30
PHIP	6	79735726	79735726	Nonsense_Mutation	SNP	C	C	T	c.(754-756)tgG>tgA	c.756G>A	p.W252*	945	26	971	46.86261	NA	0.026777	CHIP5
PHIP	6	79752631	79752631	Nonsense_Mutation	SNP	T	T	A	c.(529-531)Aaa>Taa	c.529A>T	p.K177*	906	12	918	14.686675	NA	0.013072	CHIP631
PPM1D	17	58740375	58740375	Nonsense_Mutation	SNP	G	G	A	c.(1279-1281)tGg>tAg	c.1280G>A	p.W427*	625	70	695	166.174328	NA	0.100719	CHIP586
PPM1D	17	58740546	58740546	Nonsense_Mutation	SNP	T	T	G	c.(1450-1452)tTa>tGa	c.1451T>G	p.L484*	862	80	942	191.045495	NA	0.084926	CHIP277
PPM1D	17	58740708	58740708	Nonsense_Mutation	SNP	T	T	G	c.(1612-1614)tTa>tGa	c.1613T>G	p.L538*	900	55	955	117.923965	NA	0.057592	CHIP182
PPM1D	17	58740518	58740518	Nonsense_Mutation	SNP	G	G	T	c.(1423-1425)Gaa>Taa	c.1423G>T	p.E475*	909	32	941	54.58265	NA	0.034006	CHIP573
PPM1D	17	58740603	58740603	Nonsense_Mutation	SNP	C	C	G	c.(1507-1509)tCa>tGa	c.1508C>G	p.S503*	909	29	938	60.098461	NA	0.030917	CHIP573
PPM1D	17	58740467	58740467	Nonsense_Mutation	SNP	C	C	T	c.(1372-1374)Cga>Tga	c.1372C>T	p.R458*	633	18	651	27.764967	NA	0.02765	CHIP350
PPM1D	17	58740479	58740479	Nonsense_Mutation	SNP	C	C	T	c.(1384-1386)Caa>Taa	c.1384C>T	p.Q462*	905	21	926	36.956013	NA	0.022678	CHIP586
PPM1D	17	58740668	58740668	Nonsense_Mutation	SNP	G	G	T	c.(1573-1575)Gaa>Taa	c.1573G>T	p.E525*	925	20	945	35.662692	NA	0.021164	CHIP57
PPM1D	17	58740376	58740376	Nonsense_Mutation	SNP	G	G	A	c.(1279-1281)tgG>tgA	c.1281G>A	p.W427*	726	13	739	21.362356	NA	0.017591	CHIP573
PPM1D	17	58740375	58740375	Nonsense_Mutation	SNP	G	G	A	c.(1279-1281)tGg>tAg	c.1280G>A	p.W427*	717	12	729	19.309458	NA	0.016461	CHIP573
PPM1D	17	58740705	58740718	Frame_Shift_Del	DEL	CATTAGAAGAGTCC	CATTAGAAGAGTCC	-	c.(1609-1623)acattagaagagtccfs	c.1610_1623delCATTAGAAGAGTCC	p.TLEES537fs	1324	21	1345	NA	341	0.01561338	CHIP194
PPM1D	17	58740749	58740749	Nonsense_Mutation	SNP	C	C	T	c.(1654-1656)Cga>Tga	c.1654C>T	p.R552*	913	14	927	21.21588	NA	0.015102	CHIP272
PPM1D	17	58740546	58740546	Nonsense_Mutation	SNP	T	T	A	c.(1450-1452)tTa>tAa	c.1451T>A	p.L484*	929	14	943	17.732642	NA	0.014846	CHIP73
PPM1D	17	58740498	58740498	Nonsense_Mutation	SNP	C	C	G	c.(1402-1404)tCa>tGa	c.1403C>G	p.S468*	913	13	926	22.056666	NA	0.014039	CHIP573
PPM1D	17	58740357	58740357	Splice_Site	SNP	C	C	G	c.(1261-1263)tCa>tGa	c.1262C>G	p.S421*	614	8	622	13.171981	NA	0.012862	CHIP573
PPM1D	17	58740500	58740500	Nonsense_Mutation	SNP	A	A	T	c.(1405-1407)Aaa>Taa	c.1405A>T	p.K469*	917	9	926	12.419346	NA	0.009719	CHIP573
PPM1D	17	58740809	58740809	Nonsense_Mutation	SNP	C	C	T	c.(1714-1716)Cga>Tga	c.1714C>T	p.R572*	874	8	882	12.162351	NA	0.00907	CHIP320
PPM1D	17	58740749	58740749	Nonsense_Mutation	SNP	C	C	T	c.(1654-1656)Cga>Tga	c.1654C>T	p.R552*	948	8	956	11.882799	NA	0.008368	CHIP573
PPM1D	17	58740529	58740529	Nonsense_Mutation	SNP	C	C	A	c.(1432-1434)tgC>tgA	c.1434C>A	p.C478*	930	7	937	9.672744	NA	0.007471	CHIP573
PRPF8	17	1563289	1563289	Missense_Mutation	SNP	C	C	T	c.(4792-4794)Gac>Aac	c.4792G>A	p.D1598N	855	24	879	41.311982	NA	0.027304	CHIP54
RPL11	1	24019145	24019145	Missense_Mutation	SNP	G	G	A	c.(52-54)cGc>cAc	c.53G>A	p.R18H	451	424	875	1340.04755	NA	0.01009	CHIP562
RUNX1	21	36252877	36252877	Missense_Mutation	SNP	C	C	T	c.(403-405)aGg>aAg	c.404G>A	p.R135K	877	40	917	79.657883	NA	0.043621	CHIP373
RUNX1	21	36164775	36164775	Missense_Mutation	SNP	C	C	T	c.(1099-1101)gGc>gAc	c.1100G>A	p.G367D	308	6	314	10.387951	NA	0.019108	CHIP436
RUNX1	21	36164775	36164775	Missense_Mutation	SNP	C	C	T	c.(1099-1101)gGc>gAc	c.1100G>A	p.G367D	314	6	320	8.883718	NA	0.01875	CHIP596
RUNX1	21	36164775	36164775	Missense_Mutation	SNP	C	C	T	c.(1099-1101)gGc>gAc	c.1100G>A	p.G367D	243	4	247	7.034179	NA	0.016194	CHIP590
RUNX1	21	36164775	36164775	Missense_Mutation	SNP	C	C	T	c.(1099-1101)gGc>gAc	c.1100G>A	p.G367D	578	5	583	7.121558	NA	0.008576	CHIP48
SETD2	3	47155366	47155366	Splice_Site	SNP	G	G	A	c.(4714-4716)tCg>tTg	c.4715C>T	p.S1572L	660	9	669	14.018119	NA	0.013453	CHIP93
SF3B1	2	198267359	198267359	Missense_Mutation	SNP	C	C	G	c.(1996-1998)aaG>aaC	c.1998G>C	p.K666N	698	198	896	586.790068	NA	0.220982	CHIP259
SF3B1	2	198266834	198266834	Missense_Mutation	SNP	T	T	C	c.(2098-2100)Aaa>Gaa	c.2098A>G	p.K700E	795	134	929	353.067705	NA	0.144241	CHIP305
SF3B1	2	198267491	198267491	Missense_Mutation	SNP	C	C	G	c.(1864-1866)gaG>gaC	c.1866G>C	p.E622D	902	16	918	27.163152	NA	0.017429	CHIP45
SF3B1	2	198266834	198266834	Missense_Mutation	SNP	T	T	C	c.(2098-2100)Aaa>Gaa	c.2098A>G	p.K700E	896	10	906	15.923879	NA	0.011038	CHIP344
STAG1	3	136162247	136162247	Splice_Site	SNP	C	C	A	c.e15-1			940	25	965	6.744057	NA	0.025907	CHIP239
STAG1	3	136162248	136162248	Splice_Site	SNP	T	T	A	c.e15-2			790	18	808	11.052845	NA	0.022277	CHIP307
STAG2	X	123229240	123229240	Nonsense_Mutation	SNP	C	C	T	c.(3724-3726)Cga>Tga	c.3724C>T	p.R1242*	470	46	516	106.505761	NA	0.089147	CHIP196
STAG2	X	123197045	123197045	Missense_Mutation	SNP	G	G	A	c.(1810-1812)cGa>cAa	c.1811G>A	p.R604Q	334	7	341	11.644719	NA	0.020528	CHIP386
STAT5B	17	40362213	40362213	Missense_Mutation	SNP	T	T	A	c.(1882-1884)Acc>Tcc	c.1882A>T	p.T628S	907	26	933	45.397951	NA	0.027867	CHIP190
SUZ12	17	30303572	30303572	Nonsense_Mutation	SNP	C	C	T	c.(856-858)Cga>Tga	c.856C>T	p.R286*	942	7	949	8.498681	NA	0.007376	CHIP531
TERT	5	1260675	1260675	Missense_Mutation	SNP	G	G	A	c.(2884-2886)Cgc>Tgc	c.2884C>T	p.R962C	848	20	868	36.755965	NA	0.023041	CHIP533
TET2	4	106157215	106157215	Nonsense_Mutation	SNP	C	C	T	c.(2116-2118)Cag>Tag	c.2116C>T	p.Q706*	479	474	953	1531.31125	NA	0.497377	CHIP489
TET2	4	106164085	106164086	Splice_Site	INS	-	-	T	c.e5+1			889	238	1127	NA	466	0.21118012	CHIP15
TET2	4	106156687	106156687	Nonsense_Mutation	SNP	C	C	T	c.(1588-1590)Cag>Tag	c.1588C>T	p.Q530*	762	181	943	477.173365	NA	0.191941	CHIP276
TET2	4	106156048	106156063	Frame_Shift_Del	DEL	CAGAAACCAGAACAAC	CAGAAACCAGAACAAC	-	c.(949-966)cagaaaccagaacaactafs	c.949_964delCAGAAACCAGAACAAC	p.QKPEQL317fs	878	145	1023	NA	481	0.14173998	CHIP221
TET2	4	106183007	106183007	Splice_Site	SNP	T	T	A	c.e8+2			521	34	555	64.750281	NA	0.061261	CHIP597
TET2	4	106190819	106190819	Missense_Mutation	SNP	G	G	A	c.(4096-4098)cGt>cAt	c.4097G>A	p.R1366H	867	32	899	64.62362	NA	0.035595	CHIP243
TET2	4	106197375	106197375	Missense_Mutation	SNP	A	A	C	c.(5707-5709)cAg>cCg	c.5708A>C	p.Q1903P	920	29	949	44.88985	NA	0.030558	CHIP45
TET2	4	106180841	106180841	Missense_Mutation	SNP	C	C	T	c.(3868-3870)tCa>tTa	c.3869C>T	p.S1290L	874	24	898	42.878627	NA	0.026726	CHIP77
TET2	4	106156066	106156066	Nonsense_Mutation	SNP	C	C	T	c.(967-969)Caa>Taa	c.967C>T	p.Q323*	909	24	933	40.916592	NA	0.025723	CHIP54
TET2	4	106156069	106156069	Nonsense_Mutation	SNP	C	C	T	c.(970-972)Caa>Taa	c.970C>T	p.Q324*	859	21	880	39.686328	NA	0.023864	CHIP92
TET2	4	106193892	106193892	Nonsense_Mutation	SNP	C	C	T	c.(4354-4356)Cga>Tga	c.4354C>T	p.R1452*	903	21	924	36.630996	NA	0.022727	CHIP362
TET2	4	106155363	106155363	Nonsense_Mutation	SNP	T	T	A	c.(262-264)tgT>tgA	c.264T>A	p.C88*	917	19	936	27.013072	NA	0.020299	CHIP471
TET2	4	106157788	106157788	Nonsense_Mutation	SNP	C	C	T	c.(2689-2691)Cag>Tag	c.2689C>T	p.Q897*	911	17	928	28.431199	NA	0.018319	CHIP12
TET2	4	106156048	106156048	Nonsense_Mutation	SNP	C	C	T	c.(949-951)Cag>Tag	c.949C>T	p.Q317*	889	15	904	22.730456	NA	0.016593	CHIP53
TET2	4	106193727	106193727	Missense_Mutation	SNP	A	A	G	c.(4189-4191)Act>Gct	c.4189A>G	p.T1397A	921	13	934	16.075468	NA	0.013919	CHIP312
TET2	4	106196756	106196756	Nonsense_Mutation	SNP	G	G	T	c.(5089-5091)Gga>Tga	c.5089G>T	p.G1697*	941	13	954	12.466384	NA	0.013627	CHIP623

TET2	4	106157856	106157856	Nonsense_Mutation	SNP	C	C	G	c.(2755-2757)taC>taG	c.2757C>G	p.Y919*	906	11	917	16.470858	NA	0.011996	CHIP179
TET2	4	106157527	106157527	Nonsense_Mutation	SNP	C	C	T	c.(2428-2430)Cag>Tag	c.2428C>T	p.Q810*	917	11	928	15.135785	NA	0.011853	CHIP290
TET2	4	106180775	106180775	Splice_Site	SNP	G	G	A	c.e7-1			931	11	942	11.821004	NA	0.011677	CHIP306
TET2	4	106193931	106193931	Nonsense_Mutation	SNP	C	C	T	c.(4393-4395)Cga>Tga	c.4393C>T	p.R1465*	926	10	936	14.517997	NA	0.010684	CHIP416
TP53	17	7579358	7579358	Missense_Mutation	SNP	C	C	T	c.(328-330)cGt>cAt	c.329G>A	p.R110H	487	444	931	1410.71765	NA	0.476907	CHIP121
TP53	17	7578203	7578203	Missense_Mutation	SNP	C	C	T	c.(646-648)Gtg>Atg	c.646G>A	p.V216M	803	164	967	407.392053	NA	0.169597	CHIP534
TP53	17	7577120	7577120	Missense_Mutation	SNP	C	C	T	c.(817-819)cGt>cAt	c.818G>A	p.R273H	914	51	965	108.98526	NA	0.05285	CHIP549
TP53	17	7577559	7577559	Missense_Mutation	SNP	G	G	A	c.(721-723)tCc>tTc	c.722C>T	p.S241F	895	44	939	89.916509	NA	0.046858	CHIP585
TP53	17	7577539	7577539	Missense_Mutation	SNP	G	G	A	c.(742-744)Cgg>Tgg	c.742C>T	p.R248W	868	36	904	70.107659	NA	0.039823	CHIP221
TP53	17	7577120	7577120	Missense_Mutation	SNP	C	C	T	c.(817-819)cGt>cAt	c.818G>A	p.R273H	876	31	907	59.402858	NA	0.034179	CHIP564
TP53	17	7578538	7578538	Missense_Mutation	SNP	T	T	C	c.(391-393)aAc>aGc	c.392A>G	p.N131S	911	31	942	63.048218	NA	0.032909	CHIP106
TP53	17	7578211	7578211	Missense_Mutation	SNP	C	C	T	c.(637-639)cGa>cAa	c.638G>A	p.R213Q	922	30	952	55.230635	NA	0.031513	CHIP398
TP53	17	7579377	7579377	Nonsense_Mutation	SNP	G	G	A	c.(310-312)Cag>Tag	c.310C>T	p.Q104*	888	27	915	53.458398	NA	0.029508	CHIP505
TP53	17	7577539	7577539	Missense_Mutation	SNP	G	G	A	c.(742-744)Cgg>Tgg	c.742C>T	p.R248W	895	24	919	48.210636	NA	0.026115	CHIP573
TP53	17	7577120	7577120	Missense_Mutation	SNP	C	C	A	c.(817-819)cGt>cTt	c.818G>T	p.R273L	943	20	963	37.249775	NA	0.020768	CHIP254
TP53	17	7577120	7577120	Missense_Mutation	SNP	C	C	T	c.(817-819)cGt>cAt	c.818G>A	p.R273H	928	19	947	33.040633	NA	0.020063	CHIP505
TP53	17	7577538	7577538	Missense_Mutation	SNP	C	C	T	c.(742-744)cGg>cAg	c.743G>A	p.R248Q	922	18	940	34.564431	NA	0.019149	CHIP585
TP53	17	7577545	7577545	Missense_Mutation	SNP	T	T	G	c.(736-738)Atg>Ctg	c.736A>C	p.M246L	897	16	913	26.124142	NA	0.017525	CHIP573
TP53	17	7577545	7577545	Missense_Mutation	SNP	T	T	G	c.(736-738)Atg>Ctg	c.736A>C	p.M246L	935	15	950	25.028048	NA	0.015789	CHIP553
TP53	17	7577550	7577550	Missense_Mutation	SNP	C	C	G	c.(730-732)gGc>gCc	c.731G>C	p.G244A	896	14	910	21.422764	NA	0.015385	CHIP505
TP53	17	7577108	7577108	Missense_Mutation	SNP	C	C	T	c.(829-831)tGt>tAt	c.830G>A	p.C277Y	942	13	955	20.107239	NA	0.013613	CHIP599
TP53	17	7577120	7577120	Missense_Mutation	SNP	C	C	T	c.(817-819)cGt>cAt	c.818G>A	p.R273H	966	13	979	18.494983	NA	0.013279	CHIP47
TP53	17	7578265	7578265	Missense_Mutation	SNP	A	A	G	c.(583-585)aTc>aCc	c.584T>C	p.I195T	946	12	958	18.874073	NA	0.012526	CHIP54
TP53	17	7577539	7577539	Missense_Mutation	SNP	G	G	A	c.(742-744)Cgg>Tgg	c.742C>T	p.R248W	914	11	925	18.27095	NA	0.011892	CHIP599
TP53	17	7578203	7578203	Missense_Mutation	SNP	C	C	T	c.(646-648)Gtg>Atg	c.646G>A	p.V216M	921	11	932	15.425363	NA	0.011803	CHIP41
TP53	17	7579312	7579312	Splice_Site	SNP	C	C	T	c.(373-375)acG>acA	c.375G>A	p.T125T	932	11	943	16.432387	NA	0.011665	CHIP78
TP53	17	7579538	7579538	Missense_Mutation	SNP	A	A	G	c.(148-150)aTt>aCt	c.149T>C	p.I50T	912	10	922	15.758309	NA	0.010846	CHIP442
TP53	17	7578479	7578479	Missense_Mutation	SNP	G	G	C	c.(451-453)Ccc>Gcc	c.451C>G	p.P151A	927	8	935	11.661517	NA	0.008556	CHIP199
TP53	17	7577568	7577568	Missense_Mutation	SNP	C	C	T	c.(712-714)tGt>tAt	c.713G>A	p.C238Y	915	7	922	8.934094	NA	0.007592	CHIP548
TRAF3	14	103336613	103336626	Frame_Shift_Del	DEL	CAGTGCTGGGACGC	CAGTGCTGGGACGC	-	c.(73-90)cgcagtgctgggacgccafs	c.75_88delCAGTGCTGGGACGC	p.SAGTP26fs	626	302	928	NA	491	0.32543103	CHIP294

Supplementary Table 4. Distribution of mutated genes by age groups.

Gene by age group and type.

	Frameshift	Missense	Nonsense	Splice
**30-39**				
	TRAF3	KRAS		
**40-49**				
		CBL, DNMT3A x 3, TP53 x 2	PPM1D	DNMT3A
**50-59**				
	ASXL1 x 2, DNMT3A x 3, PPM1D, TET2	DNMT3A x 7, GNB1, JAK2, KRAS x 2, RUNX1 x 3, SF3B1 x 2, TERT, TET2, TP53 x 3	ASXL1, BRCC3 x 2, DNMT3A x 7, NF1, PHIP, PPM1D x 2, SF3B1, TET2 x 4, TP53	ATM, DNMT3A x 4, SETD2, TET2
**60-69**				
	ASXL1 x 3, BRCC3, DNMT3A x 3, TET2, TP53	ASXL1, CBL, DNMT3A x 7, GNB1 x 2, JAK2 x 3, JAK3, KDM6A, KRAS x 2, PPM1D, PRPF8, SF3B1, STAG2, STAT5B, TET2 x 3, TP53 x 6	ASXL1 x 5, DNMT3A x 8, PHIP x 2, PPM1D x 4, RPL11, STAG2, TET2 x 6, TP53	ATM x 2, DNMT3A x 6, GNB1, NF1, RUNX1, STAG1 x 2, TP53 x 2
**70-79**				
		DNMT3A, KRAS, TET2, TP53	DNMT3A x 2, TET2	ASXL1, DNMT3A, TET2, TP53

Supplementary Table 5. Called somatic variants at TMN.

Gene	Chromosome	Start Position	End Position	Variant Classification	Variant Type	Reference Allele	Tumor_Seq_Allele1	Tumor_Seq_Allele2	cDNA_Change	Codon_Change	Protein Change	# Reference Reads	# Alternate Reads	Total Depth	TLOD Score	QSI_NT Score	Variant Allele Frequency	Sample
ASXL1	20	31023087	31023087	Nonsense_Mutation	SNP	C	C	T	c.2572C>T	c.(2572-2574)Cag>Tag	p.Q858*	363	309	672	925.132396	NA	0.459821	CHIP727
BCOR	X	39933387	39933405	Frame_Shift_Del	DEL	CTGGGCACCTTCGCCCCCT	CTGGGCACCTTCGCCCCCT	-	c.1194_1212delAGGGGGCGAAGGTGCCCAG	c.(1192-1212)gaagggggcgaaggtgcccagfs	p.EGGEGAQ398fs	413	20	433	NA	214	0.046189376	CHIP732
BCORL1	X	129190010	129190011	Frame_Shift_Ins	INS	-	-	C	c.5035_5036insC	c.(5035-5037)tccfs	p.S1679fs	181	36	217	NA	85	0.165898618	CHIP732
CSF3R	1	36932296	36932296	Nonsense_Mutation	SNP	G	G	A	c.2173C>T	c.(2173-2175)Cag>Tag	p.Q725*	189	26	215	57.201528	NA	0.12093	CHIP732
DNMT3A	2	25469919	25469919	Splice_Site	SNP	C	C	T		c.e9+1		332	16	348	34.468328	NA	0.045977	CHIP710
DNMT3A	2	25457161	25457161	Missense_Mutation	SNP	A	A	G	c.2726T>C	c.(2725-2727)tTt>tCt	p.F909S	447	16	463	29.161884	NA	0.034557	CHIP710
DNMT3A	2	25464578	25464578	Splice_Site	SNP	T	T	C		c.e17-2		157	132	289	335.19952	NA	0.456747	CHIP725
DNMT3A	2	25469919	25469919	Splice_Site	SNP	C	C	T		c.e9+1		177	85	262	228.952639	NA	0.324427	CHIP732
DNMT3A	2	25469919	25469919	Splice_Site	SNP	C	C	T		c.e9+1		280	71	351	186.987855	NA	0.202279	CHIP734
ETV6	12	12037484	12037484	Missense_Mutation	SNP	A	A	G	c.1115A>G	c.(1114-1116)gAt>gGt	p.D372G	278	492	770	1593.97768	NA	0.638961	CHIP727
KRAS	12	25398284	25398284	Missense_Mutation	SNP	C	C	T	c.35G>A	c.(34-36)gGt>gAt	p.G12D	822	42	864	86.024442	NA	0.048611	CHIP705
KRAS	12	25398282	25398282	Missense_Mutation	SNP	C	C	G	c.37G>C	c.(37-39)Ggc>Cgc	p.G13R	730	43	773	82.685816	NA	0.055627	CHIP723
NRAS	1	115256529	115256529	Missense_Mutation	SNP	T	T	A	c.182A>T	c.(181-183)cAa>cTa	p.Q61L	755	62	817	140.691431	NA	0.075887	CHIP727
PPM1D	17	58740375	58740375	Nonsense_Mutation	SNP	G	G	A	c.1280G>A	c.(1279-1281)tGg>tAg	p.W427*	459	25	484	53.118569	NA	0.051653	CHIP704
PPM1D	17	58740498	58740498	Nonsense_Mutation	SNP	C	C	G	c.1403C>G	c.(1402-1404)tCa>tGa	p.S468*	831	16	847	29.115405	NA	0.01889	CHIP704
PPM1D	17	58740375	58740375	Nonsense_Mutation	SNP	G	G	A	c.1280G>A	c.(1279-1281)tGg>tAg	p.W427*	468	21	489	41.033696	NA	0.042945	CHIP705
PPM1D	17	58740708	58740708	Nonsense_Mutation	SNP	T	T	G	c.1613T>G	c.(1612-1614)tTa>tGa	p.L538*	842	9	851	13.085652	NA	0.010576	CHIP734
RUNX1	21	36252877	36252877	Missense_Mutation	SNP	C	C	T	c.404G>A	c.(403-405)aGg>aAg	p.R135K	845	40	885	81.595481	NA	0.045198	CHIP710
RUNX1	21	36164775	36164775	Missense_Mutation	SNP	C	C	T	c.1100G>A	c.(1099-1101)gGc>gAc	p.G367D	120	4	124	7.742208	NA	0.032258	CHIP716
RUNX1	21	36252877	36252877	Missense_Mutation	SNP	C	C	T	c.404G>A	c.(403-405)aGg>aAg	p.R135K	207	236	443	714.698499	NA	0.532731	CHIP732
RUNX1	21	36252877	36252877	Missense_Mutation	SNP	C	C	T	c.404G>A	c.(403-405)aGg>aAg	p.R135K	603	174	777	484.469099	NA	0.223938	CHIP734
SF3B1	2	198266834	198266834	Missense_Mutation	SNP	T	T	C	c.2098A>G	c.(2098-2100)Aaa>Gaa	p.K700E	598	45	643	96.817643	NA	0.069984	CHIP715
SF3B1	2	198266834	198266834	Missense_Mutation	SNP	T	T	C	c.2098A>G	c.(2098-2100)Aaa>Gaa	p.K700E	663	67	730	153.140966	NA	0.091781	CHIP716
SRSF2	17	74732959	74732959	Missense_Mutation	SNP	G	G	T	c.284C>A	c.(283-285)cCc>cAc	p.P95H	155	173	328	541.3371	NA	0.527439	CHIP727
STAG2	X	123220476	123220476	Nonsense_Mutation	SNP	C	C	T	c.3133C>T	c.(3133-3135)Cga>Tga	p.R1045*	348	24	372	52.066567	NA	0.064516	CHIP727
STAG2	X	123179013	123179013	Splice_Site	SNP	G	G	T		c.e8-1		77	130	207	432.374854	NA	0.628019	CHIP727
STAG2	X	123195141	123195141	Nonsense_Mutation	SNP	G	G	A	c.(1483-1485)tGg>tAg	c.1484G>A	p.W495*	526	8	534	11.553915	NA	0.014981	CHIP727
TET2	4	106196756	106196756	Nonsense_Mutation	SNP	G	G	T	c.5089G>T	c.(5089-5091)Gga>Tga	p.G1697*	906	10	916	10.478309	NA	0.010917	CHIP703
TET2	4	106196756	106196756	Nonsense_Mutation	SNP	G	G	T	c.5089G>T	c.(5089-5091)Gga>Tga	p.G1697*	897	11	908	15.63225	NA	0.012115	CHIP704
TET2	4	106156048	106156063	Frame_Shift_Del	DEL	CAGAAACCAGAACAAC	CAGAAACCAGAACAAC	-	c.949_964delCAGAAACCAGAACAAC	c.(949-966)cagaaaccagaacaactafs	p.QKPEQL317fs	711	87	798	NA	425	0.109022556	CHIP719
TET2	4	106156048	106156063	Frame_Shift_Del	DEL	CAGAAACCAGAACAAC	CAGAAACCAGAACAAC	-	c.949_964delCAGAAACCAGAACAAC	c.(949-966)cagaaaccagaacaactafs	p.QKPEQL317fs	529	143	672	NA	495	0.212797619	CHIP723
TET2	4	106180796	106180796	Missense_Mutation	SNP	G	G	A	c.3824G>A	c.(3823-3825)gGg>gAg	p.G1275E	621	12	633	22.227844	NA	0.018957	CHIP734
TP53	17	7578538	7578538	Missense_Mutation	SNP	T	T	G	c.392A>C	c.(391-393)aAc>aCc	p.N131T	493	31	524	62.846422	NA	0.05916	CHIP704
TP53	17	7578268	7578268	Missense_Mutation	SNP	A	A	T	c.581T>A	c.(580-582)cTt>cAt	p.L194H	712	165	877	456.042022	NA	0.188141	CHIP704
TP53	17	7578260	7578260	Missense_Mutation	SNP	C	C	G	c.589G>C	c.(589-591)Gtg>Ctg	p.V197L	872	7	879	6.61315	NA	0.007964	CHIP705
TP53	17	7578268	7578268	Missense_Mutation	SNP	A	A	T	c.581T>A	c.(580-582)cTt>cAt	p.L194H	609	268	877	805.286123	NA	0.305587	CHIP705
TP53	17	7578190	7578190	Missense_Mutation	SNP	T	T	C	c.659A>G	c.(658-660)tAt>tGt	p.Y220C	697	143	840	372.682362	NA	0.170238	CHIP712
TP53	17	7574035	7574035	Splice_Site	SNP	T	T	A		c.e10-2		584	98	682	252.031588	NA	0.143695	CHIP712
TP53	17	7577539	7577539	Missense_Mutation	SNP	G	G	A	c.742C>T	c.(742-744)Cgg>Tgg	p.R248W	578	18	596	34.21122	NA	0.030201	CHIP719
TP53	17	7578190	7578190	Missense_Mutation	SNP	T	T	C	c.659A>G	c.(658-660)tAt>tGt	p.Y220C	264	93	357	256.965937	NA	0.260504	CHIP721
TP53	17	7574035	7574035	Splice_Site	SNP	T	T	A		c.e10-2		258	123	381	334.505065	NA	0.322835	CHIP721
TP53	17	7577539	7577539	Missense_Mutation	SNP	G	G	A	c.742C>T	c.(742-744)Cgg>Tgg	p.R248W	595	37	632	84.207553	NA	0.058544	CHIP723
TP53	17	7577120	7577120	Missense_Mutation	SNP	C	C	T	c.818G>A	c.(817-819)cGt>cAt	p.R273H	863	16	879	26.762185	NA	0.018203	CHIP723
TP53	17	7577094	7577094	Missense_Mutation	SNP	G	G	C	c.844C>G	c.(844-846)Cgg>Ggg	p.R282G	637	234	871	562.412349	NA	0.268657	CHIP723
TP53	17	7578291	7578291	Splice_Site	SNP	T	T	G		c.e6-2		564	217	781	577.821598	NA	0.277849	CHIP723
TP53	17	7577570	7577570	Missense_Mutation	SNP	C	C	T	c.711G>A	c.(709-711)atG>atA	p.M237I	167	111	278	326.310947	NA	0.399281	CHIP725
TP53	17	7578508	7578508	Missense_Mutation	SNP	C	C	T	c.422G>A	c.(421-423)tGc>tAc	p.C141Y	162	137	299	420.223452	NA	0.458194	CHIP725
TP53	17	7577509	7577509	Missense_Mutation	SNP	C	C	T	c.772G>A	c.(772-774)Gaa>Aaa	p.E258K	466	80	546	157.113464	NA	0.14652	CHIP726
TP53	17	7577539	7577539	Missense_Mutation	SNP	G	G	A	c.742C>T	c.(742-744)Cgg>Tgg	p.R248W	327	90	417	241.186773	NA	0.215827	CHIP732
TP53	17	7578440	7578440	Missense_Mutation	SNP	T	T	C	c.490A>G	c.(490-492)Aag>Gag	p.K164E	297	93	390	226.298155	NA	0.238462	CHIP733
TP53	17	7577539	7577539	Missense_Mutation	SNP	G	G	A	c.742C>T	c.(742-744)Cgg>Tgg	p.R248W	619	40	659	92.116068	NA	0.060698	CHIP734
TP53	17	7577022	7577022	Nonsense_Mutation	SNP	G	G	A	c.916C>T	c.(916-918)Cga>Tga	p.R306*	534	408	942	1255.76526	NA	0.433121	CHIP741
TP53	17	7577498	7577498	Splice_Site	SNP	C	C	T		c.e7+1		422	333	755	1033.46989	NA	0.44106	CHIP741
TP53	17	7578454	7578454	Missense_Mutation	SNP	G	G	A	c.476C>T	c.(475-477)gCc>gTc	p.A159V	262	262	524	815.094565	NA	0.5	CHIP742

Supplementary Table 6. Sequential samples of patients who developed a TMN following ASCT. Mutations reported in red are those that were force-called.

	Diagnosis		Premobilization		ASCT		TMN			2nd Time Point	
Patient	Mutations	VAF	Mutations	VAF	Mutations	VAF	TMN	Mutations	VAF	Mutations	VAF
CHIP506	NONE<br>TP53 p.M237I<br>DNMT3A Splice (25464578: T, C)	 0.004831<br>0.001603	NONE<br>TP53 p.C141Y<br>DNMT3A Splice (25464578: T, C)	 0.002786<br>0.002949	NONE<br>TP53 p.C141Y<br>DNMT3A Splice (25464578: T, C)	 0.001085<br>0.002176	MDS	TP53 p.C141Y<br>TP53 p.M237I<br>DNMT3A Splice (25464578: T, C)	0.458194<br>0.399281<br>0.456747		
CHIP623			TET2 p.G1697*<br>PPM1D p.W427*<br>PPM1D p.S468*<br>TP53 p.N131T	0.010917<br>0.003846<br>0.00114<br>0.002288	TET2 p.G1697*<br>PPM1D p.W427*	0.011473<br>0.006281	MDS	TET2 p.G1697*<br>PPM1D p.S468*<br>PPM1D p.W427*<br>TP53 p.L194H<br>TP53 p.N131T	0.013245<br>0.01889<br>0.051653<br>0.192346<br>0.05916	TET2 p.G1697*<br>PPM1D p.S468*<br>PPM1D p.W427*<br>TP53 p.L194H<br>TP53 p.V197L<br>KRAS p.G12D	0.01295<br>0.01889<br>0.042945<br>0.303571<br>0.00796<br>0.048611
CHIP373			RUNX1 p.R135K<br>DNMT3A Splice (25469919: C, T)<br>DNMT3A p.F909S<br>TP53 p.R248W	0.047418<br>0.045977<br>0.034557<br>0.001294	RUNX1 p.R135K<br>DNMT3A Splice (25469919: C, T)<br>DNMT3A p.F909S	0.04738401<br>0.04973357<br>0.023845	MDS	RUNX p.R135K<br>DNMT3A Splice (25469919: C, T)<br>TP53 p.R248W<br>PPM1D p.L538*<br>TET2 p.G1275E	0.223938<br>0.202279<br>0.060698<br>0.010576<br>0.018957	RUNX1 p.R135K<br>DNMT3A Splice (25469919: C, T)<br>TP53 p.R248W<br>BCOR p.EGGEGAQ398fs<br>BCORL1 p.S1679fs<br>CSF3R p.Q725*	0.532731<br>0.324427<br>0.215827<br>0.0461893<br>8<br>0.1658986<br>2<br>0.12093
CHIP473			NONE<br>TP53 Splice (7574035: T, A)	 0.001499	NONE<br>TP53 Splice (7574035: T, A)	 0.001078	MDS	TP53 p.Y220C<br>TP53 Splice (7574035: T, A)	0.260504<br>0.322835	TP53 p.Y220C<br>TP53 Splice (7574035: T, A)	0.170238<br>0.143695
CHIP221			TET2 p.QKPEQL317fs<br>TP53 p.R248W<br>TP53 Splice (7578291: T, G)	0.109022<br>0.030201<br>0.001264	TET2 p.QKPEQL317fs<br>TP53 p.R248W	0.14173998<br>0.039823	MDS	KRAS p.G13R<br>TET2 p.QKPEQL317fs<br>TP53 p.R248W<br>TP53 p.R273H<br>TP53 p.R282G<br>TP53 Splice (7578291: T, G)	0.055627<br>0.212797<br>0.058544<br>0.018203<br>0.268657<br>0.277849		
CHIP606			NONE		NONE		MDS	TP53 p.K164E	0.238462		
CHIP282			NONE<br>TP53 p.E258K	 0.001845	NONE		MDS	TP53 p.E258K	0.14652		
CHIP36					ASXL1 p.Q858*	0.045359	MDS	ASXL1 p.Q858*<br>ETV6 p.D372G<br>NRAS p.Q61L<br>STAG2 p.R1045*<br>STAG2 p.W495*<br>SRSF2 p.N1487fs<br>STAG2 Splice (123179013: G, T)	0.459821<br>0.638961<br>0.07411<br>0.064551<br>0.014981<br>0.011422<br>0.628019		
CHIP241					NONE<br>TP53 p.R306*<br>TP53 Splice (7577498: C, T)	 0.001054<br>0.002278	AML	TP53 p.R306*<br>TP53 Splice (7577498: C, T)	0.433121<br>0.44106		
CHIP268					NONE		AML	TP53 p.A159V	0.5		
CHIP414			NONE		NONE		AML	NONE		NONE	
CHIP472			NONE		NONE		MDS	Sample not available		NONE	
CHIP336					NONE		AML	NONE			
CHIP616					NONE		MDS	NONE			

Supplementary Table 7. Hematologic and immunologic parameters of patients at diagnosis.

			CHIP			
	**Total**		**No**	**Yes**		***p-value***
	**n = 629 (%)**		**n = 493 (78)**	**n = 136 (22)**		
**Hemoglobin**						
Median (range)	11.20 (3.00 - 16.60)		11.20 (3.00 - 16.60)	11.30 (6.80 - 15.80)		*0.40^*
Missing	*65 (10)*		*46 (9)*	*19 (14)*		
**Hematocrit**						
Median (range)	32.80 (12.00 - 49.70)		32.60 (12.00 - 49.70)	33.10 (18.20 - 47.40)		*0.35^*
Missing	*72 (11)*		*52 (11)*	*20 (15)*		
**RBC**						
Median (range)	3.60 (0.52 - 10.30)		3.59 (0.52 - 10.30)	3.70 (2.03 - 5.85)		*0.8^*
Missing	*176 (28)*		*137 (28)*	*39 (29)*		
**WBC**						
Median (range)	5.95 (1.00 - 21.48)		5.90 (1.90 - 21.48)	6.05 (1.00 - 20.40)		*0.6^*
Missing	*89 (14)*		*65 (13)*	*24 (18)*		
**MCV**						
Median (range)	92.25 (60.70 - 111.00)		92.00 (60.70 - 109.10)	92.60 (65.60 - 111.00)		*0.39^*
Missing	*167 (27)*		*135 (27)*	*32 (24)*		
**ANC**						
Median (range)	3.500 (0.340 - 19.330)		3.500 (0.400 - 19.330)	3.460 (0.340 - 15.500)		*> 0.9^*
Missing	*214 (34)*		*169 (34)*	*45 (33)*		
**Platelets**						
Median (range)	229.0 (20.0 - 1014.0)		228.5 (24.0 - 1014.0)	231.0 (20.0 - 757.0)		*0.8^*
Missing	*85 (14)*		*63 (13)*	*22 (16)*		
**IgG**						
Median (range)	4248.0 (280.0 - 13700.0)		4260.0 (280.0 - 13500.0)	4248.0 (470.0 - 13700.0)		*0.8^*
Missing	*321 (51)*		*252 (51)*	*69 (51)*		
**IgA**						
Median (range)	2670 (15 - 8620)		2735 (15 - 8620)	2561 (610 - 8140)		*0.7^*
Missing	*510 (81)*		*397 (81)*	*113 (83)*		
**Kappa**						
Median (range)	148.00 (1.46 - 22000.00)		148.00 (2.06 - 22000.00)	255.00 (1.46 - 11200.00)		*> 0.9^*

Missing	*464 (74)*		*361 (73)*	*103 (76)*		
**Lambda**						
Median (range)	532.00 (0.18 - 18000.00)		550.00 (0.18 - 18000.00)	458.50 (4.74 - 7570.00)		*0.8*^
Missing	*540 (86)*		*426 (86)*	*114 (84)*		
**K/L Ratio**						
Median (range)	8.2 (0.0 - 6940.0)		11.1 (0.0 - 6940.0)	6.2 (0.0 - 2716.8)		*0.9*^
Missing	*385 (61)*		*305 (62)*	*80 (59)*		
**β_2M**						
Median (range)	3.300 (0.800 - 32.300)		3.210 (0.800 - 32.300)	3.590 (1.200 - 21.000)		*0.090*^
Missing	*231 (37)*		*181 (37)*	*50 (37)*		
**M-spike**						
Median (range)	2.22 (0.00 - 16.90)		2.23 (0.00 - 16.90)	2.15 (0.00 - 8.60)		*0.45*^
Missing	*178 (28)*		*149 (30)*	*29 (21)*		

^Wilcoxon rank-sum test (two-sided)

Supplementary Table 8. Hematologic and immunologic parameters of patients post induction.

			CHIP			
	**Total**		**No**	**Yes**		***p-value***
	**n = 629 (%)**		**n = 493 (78)**	**n = 136 (22)**		
**Hemoglobin**						
Median (range)	10.2 (7.4 - 14.2)		10.2 (7.5 - 14.2)	10.1 (7.4 - 14.0)		*0.48^*
Missing	*1 (0)*		*1 (0)*	-		
**Hematocrit**						
Median (range)	29.70 (5.62 - 43.00)		29.65 (22.90 - 43.00)	29.80 (5.62 - 39.70)		*0.46^*
Missing	*1 (0)*		*1 (0)*	-		
**RBC**						
Median (range)	3.31 (2.24 - 5.36)		3.32 (2.24 - 4.87)	3.29 (2.48 - 5.36)		*> 0.9^*
Missing	*1 (0)*		*1 (0)*	-		
**WBC**						
Median (range)	4.20 (0.70 - 13.03)		4.17 (1.17 - 13.03)	4.32 (0.70 - 12.11)		*0.33^*
Missing	*1 (0)*		*1 (0)*	-		
**MCV**						
Median (range)	90.1 (63.8 - 109.6)		89.9 (67.6 - 109.6)	90.5 (63.8 - 105.5)		*0.27^*
Missing	*2 (0)*		*2 (0)*	-		
**ANC**						
Median (range)	3.270 (0.060 - 10.920)		3.260 (0.060 - 10.920)	3.395 (0.110 - 9.720)		*0.41^*
Missing	*54 (9)*		*42 (9)*	*12 (9)*		
**Platelets**						
Median (range)	224 (4 - 682)		225 (4 - 682)	222 (62 - 682)		*0.8^*
Missing	*1 (0)*		*1 (0)*	-		
**IgG**						
Median (range)	1004 (137 - 7650)		990 (137 - 5500)	1030 (284 - 7650)		*0.19^*
Missing	*277 (44)*		*218 (44)*	*59 (43)*		
**IgA**						
Median (range)	168 (16 - 3230)		145 (16 - 2420)	207 (26 - 3230)		*0.9^*
Missing	*503 (80)*		*392 (80)*	*111 (82)*		
**Kappa**						
Median (range)	13.60 (1.07 - 3800.00)		13.40 (1.07 - 3800.00)	14.75 (1.56 - 1880.00)		*0.17^*

Missing	*334 (53)*		*254 (52)*	*80 (59)*		
**Lambda**						
Median (range)	18.00 (1.10 - 3670.00)		14.90 (1.10 - 1490.00)	30.65 (1.10 - 3670.00)		*0.25*^
Missing	*487 (77)*		*389 (79)*	*98 (72)*		
**K/L Ratio**						
Median (range)	1.0 (0.0 - 3454.5)		1.0 (0.0 - 3454.5)	1.1 (0.0 - 1182.4)		*0.24*^
Missing	*178 (28)*		*140 (28)*	*38 (28)*		
**β_2M**						
Median (range)	2.00 (0.80 - 23.70)		2.00 (0.80 - 23.70)	2.30 (1.00 - 13.90)		*0.008*^
Missing	*33 (5)*		*25 (5)*	*8 (6)*		
**M-spike**						
Median (range)	0.21 (0.00 - 4.84)		0.20 (0.00 - 3.92)	0.33 (0.00 - 4.84)		*0.36*^
Missing	*24 (4)*		*21 (4)*	*3 (2)*		

^Wilcoxon rank-sum test (two-sided)

Supplementary Table 9. Absolute and percentage change in immunologic parameters of patients between diagnosis and post induction.

			CHIP			
	Total		No	Yes		*p-value*
	n = 629 (%)		n = 493 (78)	n = 136 (22)		
**Bone Marrow Involvement**						
Median (range)	-40 (-100 - 80)		-42 (-100 - 60)	-35 (-99 - 80)		*0.15*^
Missing	*97 (15)*		*82 (17)*	*15 (11)*		
**Bone Marrow Involvement (%)**						
Median (range)	-87 (-100 - 1600)		-88 (-100 - 1150)	-84 (-100 - 1600)		*0.11*^
Missing	*104 (17)*		*88 (18)*	*16 (12)*		
**IgA**						
Median (range)	-2340 (-8528 - 1832)		-2371 (-8528 - 1832)	-2176 (-6991 - - 501)		*0.7*^
Missing	*513 (82)*		*400 (81)*	*113 (83)*		
**IgA (%)**						
Median (range)	-91 (-100 - 312)		-91 (-100 - 312)	-89 (-99 - -56)		*0.9*^
Missing	*513 (82)*		*400 (81)*	*113 (83)*		
**IgG**						
Median (range)	-2814 (-11833 - 2734)		-2829 (-11833 - 2734)	-2704 (-10446 - 700)		*0.35*^
Missing	*326 (52)*		*257 (52)*	*69 (51)*		
**IgG (%)**						
Median (range)	-71 (-98 - 283)		-71 (-98 - 283)	-70 (-96 - 149)		*0.19*^
Missing	*326 (52)*		*257 (52)*	*69 (51)*		
**Kappa**						
Median (range)	-97 (-13987 - 2138)		-85 (-13987 - 2138)	-123 (-11194 - 253)		*0.8*^
Missing	*473 (75)*		*368 (75)*	*105 (77)*		
**Kappa (%)**						
Median (range)	-86 (-100 - 2096)		-85 (-100 - 2096)	-89 (-100 - 275)		*0.9*^
Missing	*473 (75)*		*368 (75)*	*105 (77)*		
**Lambda**						
Median (range)	-338 (-17988 - 2000)		-356 (-17988 - 883)	-325 (-7410 - 2000)		*0.6*^
Missing	*545 (87)*		*429 (87)*	*116 (85)*		
**Lambda (%)**						

Median (range)	-88 (-100 - 19678)		-88 (-100 - 19678)	-87 (-100 - 120)		*0.7*^
Missing	*545 (87)*		*429 (87)*	*116 (85)*		
**K/L Ratio**						
Median (range)	-5 (-6659 - 763)		-5 (-6659 - 763)	-1 (-2416 - 68)		*> 0.9*^
Missing	*399 (63)*		*315 (64)*	*84 (62)*		
**K/L Ratio (%)**						
Median (range)	-62 (-100 - 497903)		-62 (-100 - 497903)	-61 (-100 - 46233)		*0.8*^
Missing	*400 (64)*		*316 (64)*	*84 (62)*		
**β_2M**						
Median (range)	-1 (-28 - 12)		-1 (-28 - 12)	-1 (-17 - 9)		*0.7*^
Missing	*244 (39)*		*191 (39)*	*53 (39)*		
**β_2M (%)**						
Median (range)	-35 (-92 - 240)		-37 (-92 - 240)	-30 (-85 - 196)		*0.40*^
Missing	*244 (39)*		*191 (39)*	*53 (39)*		
**M-spike**						
Median (range)	-1 (-16 - 2)		-2 (-16 - 2)	-1 (-8 - 0)		*0.13*^
Missing	*192 (31)*		*161 (33)*	*31 (23)*		
**M-spike (%)**						
Median (range)	-84 (-100 - 218)		-86 (-100 - 218)	-76 (-100 - -2)		*0.008*^
Missing	*257 (41)*		*205 (42)*	*52 (38)*		

^Wilcoxon rank-sum test (two-sided)

SUPPLEMENTARY FIGURES

Supplementary Figure 1. Study workflow.

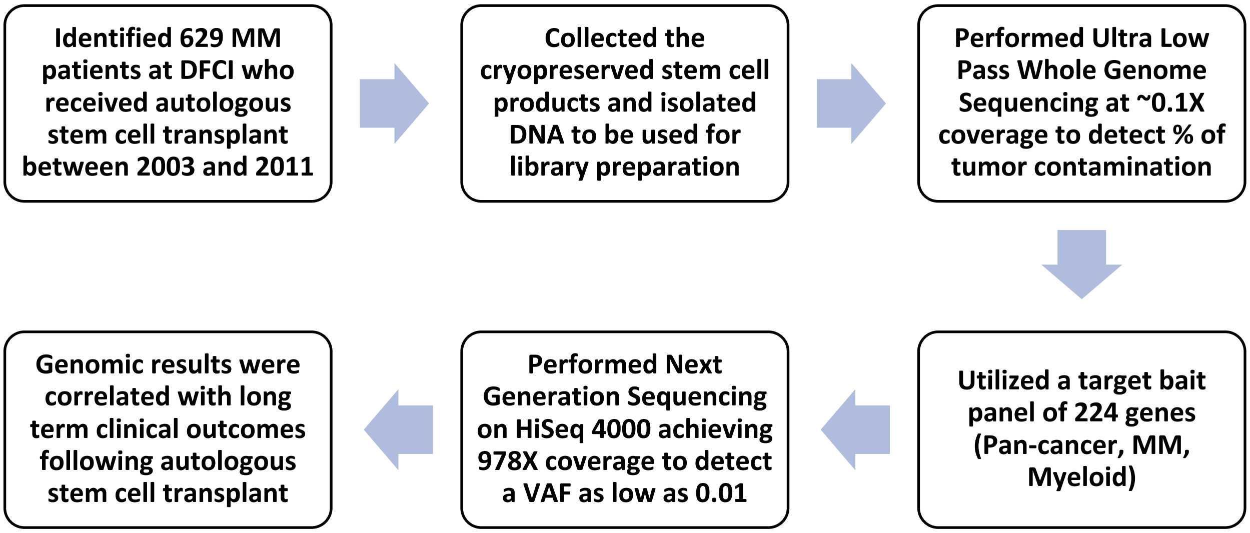

Supplementary Figure 2. Mutational properties. (A) The maximum VAF attained by each of the 136 patients with CHIP. (B) Distribution of VAF among different variants. (C) Distribution of the types of single-nucleotide base-pair changes seen in all detected mutations.

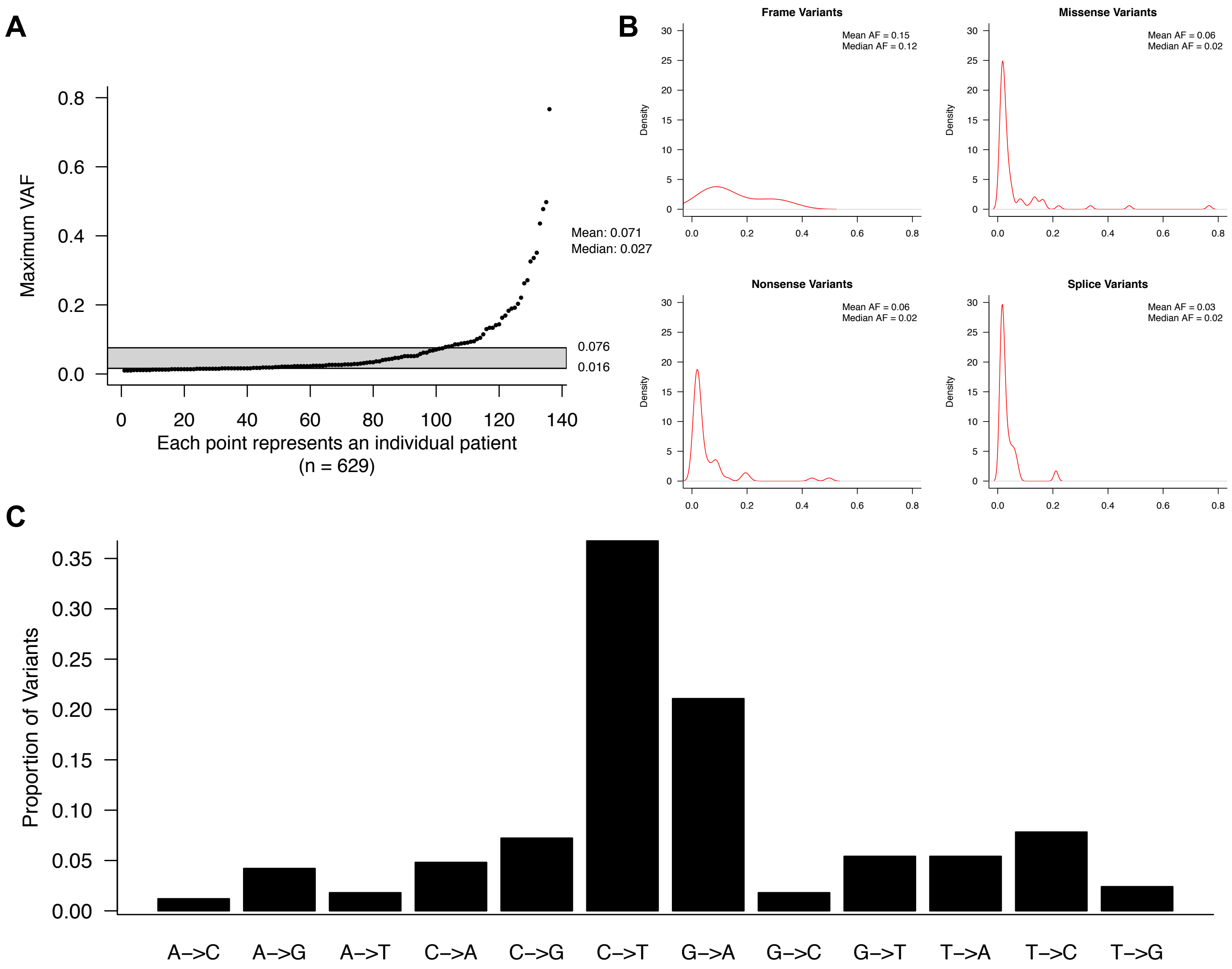

Supplementary Figure 3. Comparison of hematologic parameters of patients with CHIP vs. patients without CHIP at diagnosis. The CHIP variable contains all genes, except for *DNMT3A*, *TET2*, *TP53*, *ASXL1* and *PPM1D*. This data is derived from all 629 patients. Data are represented as boxplots where the middle line is the median, the lower and upper hinges correspond to the first and third quartiles, the upper whisker extends from the hinge to the largest value no further than 1.5 × IQR from the hinge (where IQR is the inter-quartile range) and the lower whisker extends from the hinge to the smallest value at most 1.5 × IQR of the hinge, while data beyond the end of the whiskers are outlying points that are plotted individually. Wilcoxon rank-sum p-values (two-sided, Benjamini-Hochberg correction) are stated below each variable.

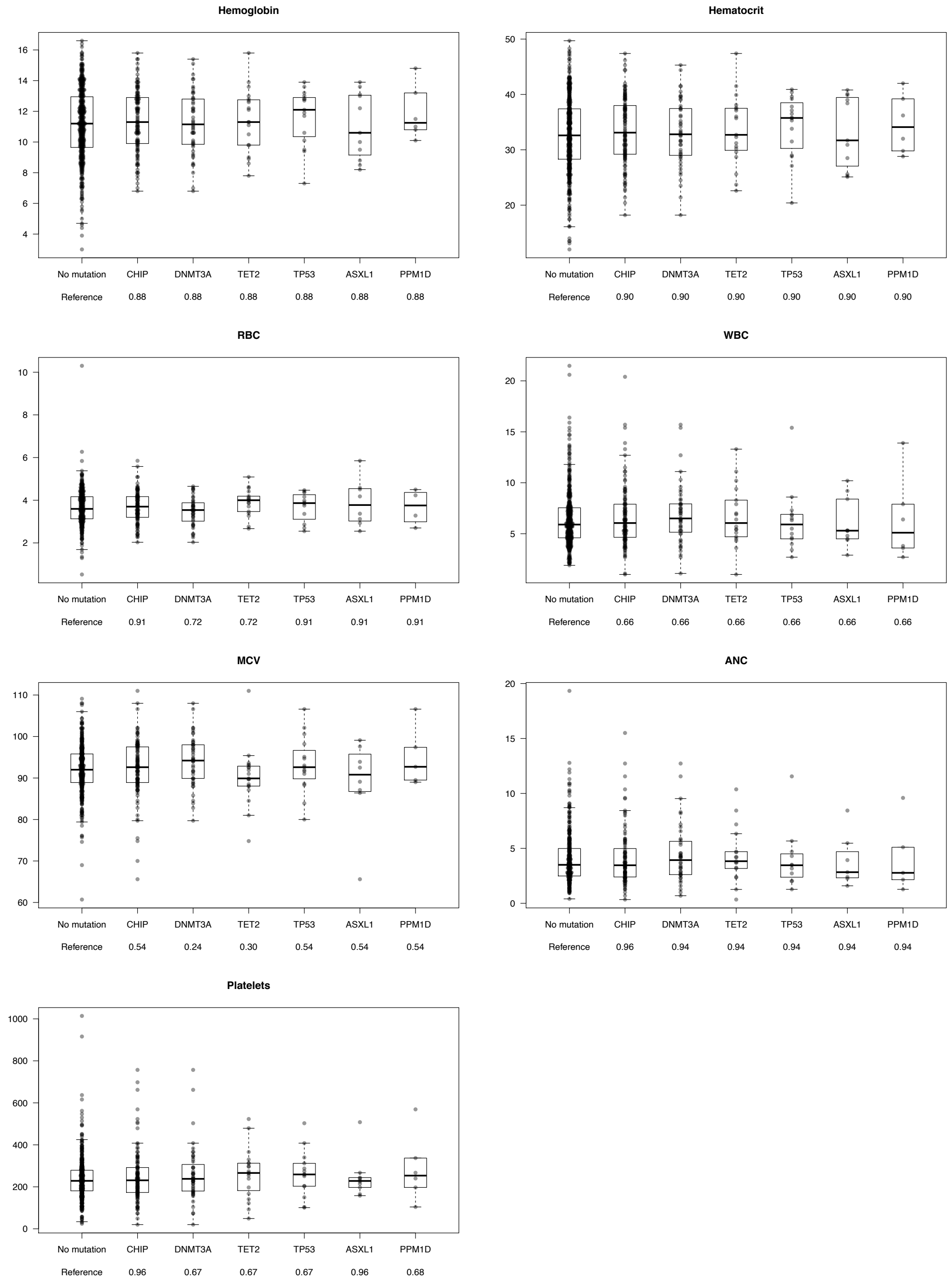

Supplementary Figure 4. Comparison of hematologic parameters of patients with CHIP vs. patients without CHIP post induction. The CHIP variable contains all genes, except for the DNMT3A, TET2, TP53, ASXL1 and PPM1D. This data is derived from all 629 patients. Data are represented as boxplots where the middle line is the median, the lower and upper hinges correspond to the first and third quartiles, the upper whisker extends from the hinge to the largest value no further than 1.5 × IQR from the hinge (where IQR is the inter-quartile range) and the lower whisker extends from the hinge to the smallest value at most 1.5 × IQR of the hinge, while data beyond the end of the whiskers are outlying points that are plotted individually. Wilcoxon rank-sum p-values (two-sided, Benjamini-Hochberg correction) are stated below each variable.

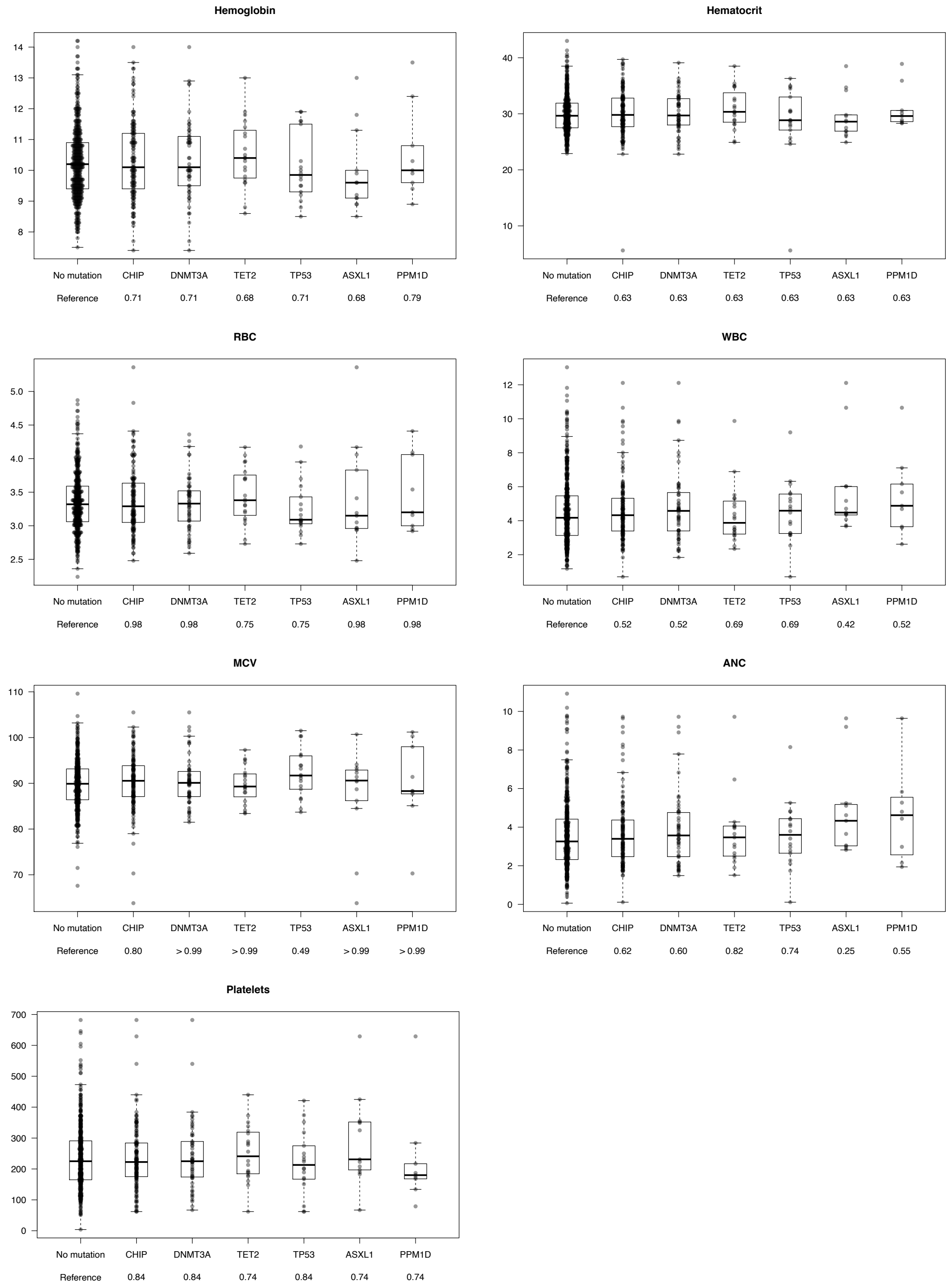

Supplementary Figure 5. Effect of number of CHIP mutations on OS and PFS. (A) OS and (B) PFS among patients with 1 CHIP mutation versus having more than 1 CHIP mutation versus those without CHIP. (C) OS and (D) PFS among patients who did not receive IMiD maintenance with respect to number of CHIP mutations. (E) OS and (F) PFS among patients who received IMiD maintenance with respect to number of CHIP mutations. Overall and pairwise two-sided log-rank p-values are shown unadjusted for multiple testing.

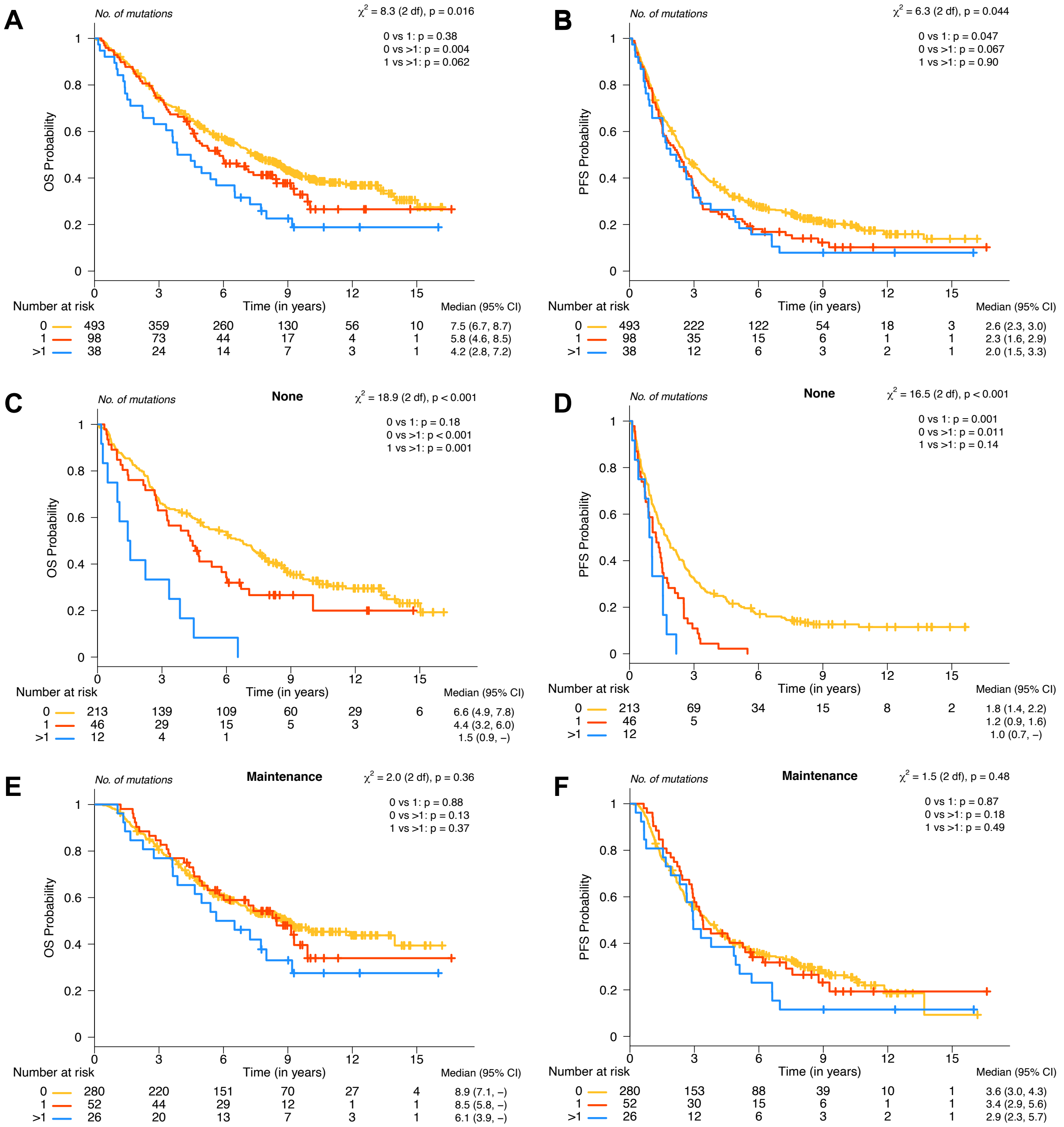

Supplementary Figure 6. OS and PFS of patients divided into IMiD, non-IMiD and None. (A) OS and (B) PFS among all patients. OS among those with CHIP (C) and those without CHIP (D). PFS among those with CHIP (E) and those without CHIP (F). Overall and pairwise two-sided log-rank p-values are shown unadjusted for multiple testing.

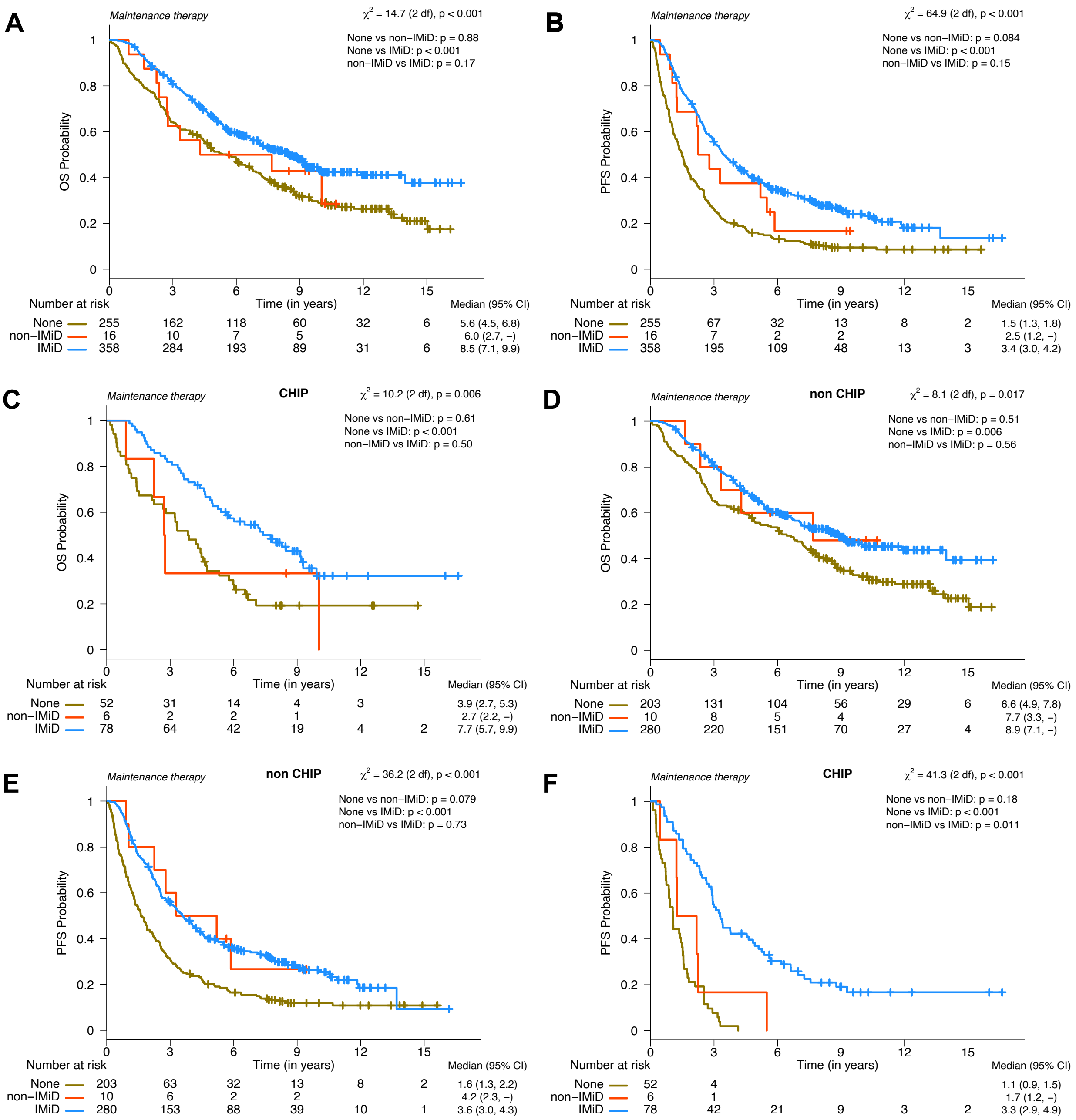

Supplementary Figure 7. Multivariable cox regression model of CHIP and IMiD interaction. (A) OS and (B) PFS models for all 629 patients after stratifying by age, ISS and number of lines of therapy prior to ASCT to investigate the effect of CHIP and IMiD maintenance on outcome and their interaction. A significant non-additive interaction exists between CHIP and IMiD maintenance. Two-sided Wald p-values are shown for each model coefficient with significant effects displayed in red. Exact p-values: A: 0.008452526, 0.00478633 and 0.178680402; B: $2.52914x10^{-6}$, $1.25518x10^{-8}$ and 0.002199723. HR: Hazard Ratio; LCI: Lower Confidence Interval; UCI: Upper Confidence Interval.

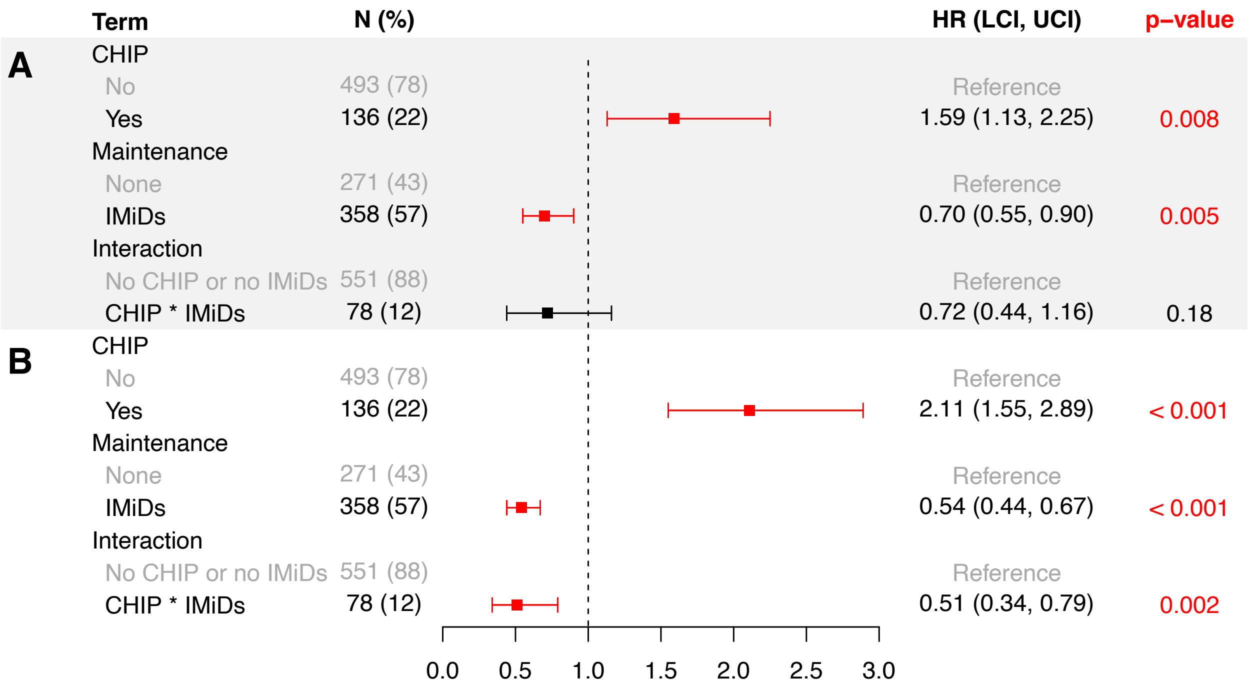

Supplementary Figure 8. PFS and OS of patients with DNMT3A and TET2 mutations with respect to IMiD maintenance. OS and PFS of patients with a DNMT3A mutation versus those with a non-DNMT3A mutation and CHIP-negative patients among those who did not receive IMiD maintenance (A-B) and those who did receive (C-D). OS and PFS among patients with a TET2 mutation and no maintenance (E-F) and patients receiving IMiD maintenance (G-H). Overall and pairwise two-sided log-rank p-values are shown unadjusted for multiple testing. MUT: studied mutation; WT: other CHIP mutations.

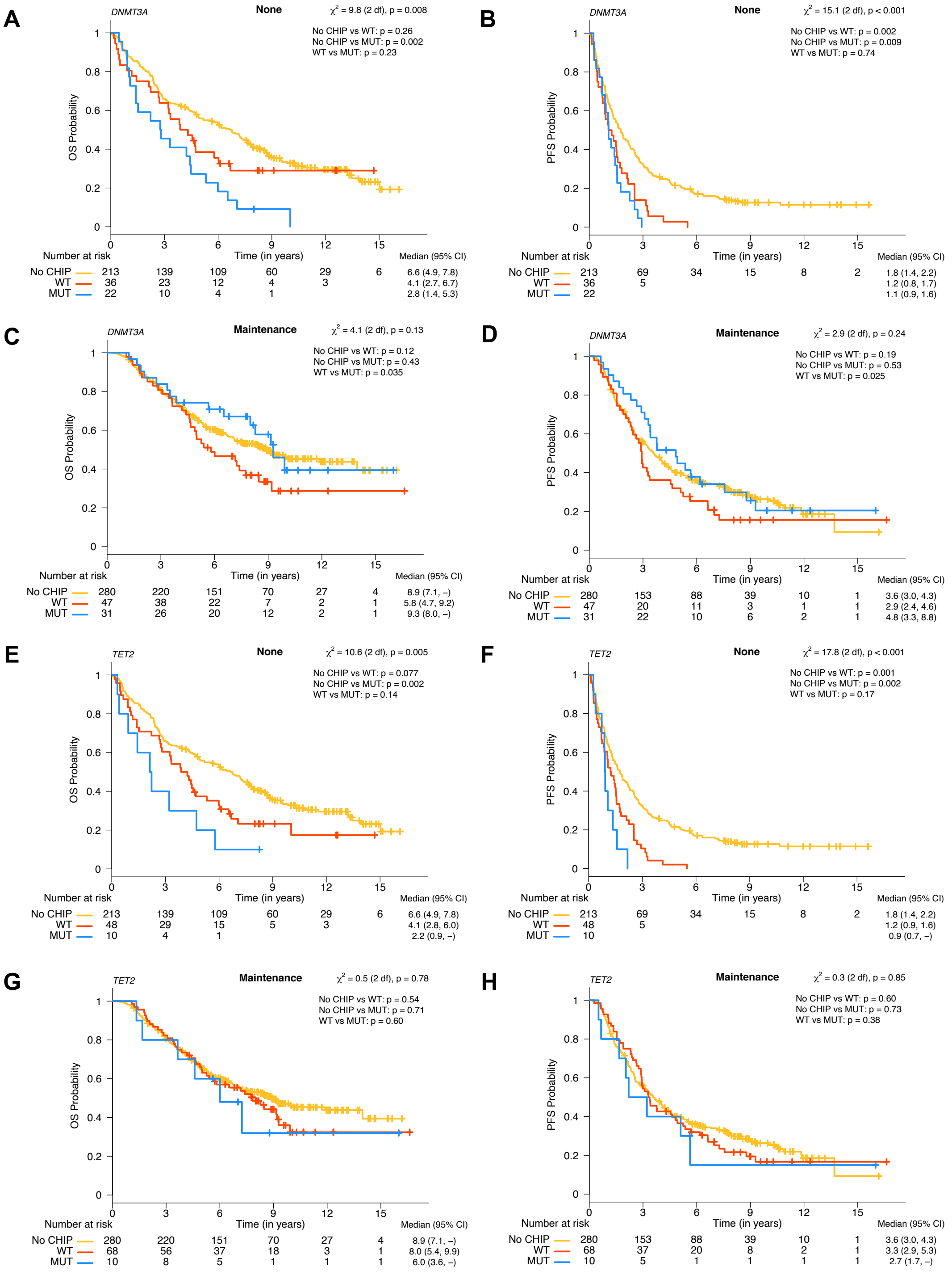

Supplementary Figure 9. OS and PFS of patients with respect to the p.R882 DNMT3A mutation. (A) OS and (B) PFS among patients who are not on IMiD maintenance. Overall and pairwise two-sided log-rank p-values are shown unadjusted for multiple testing.

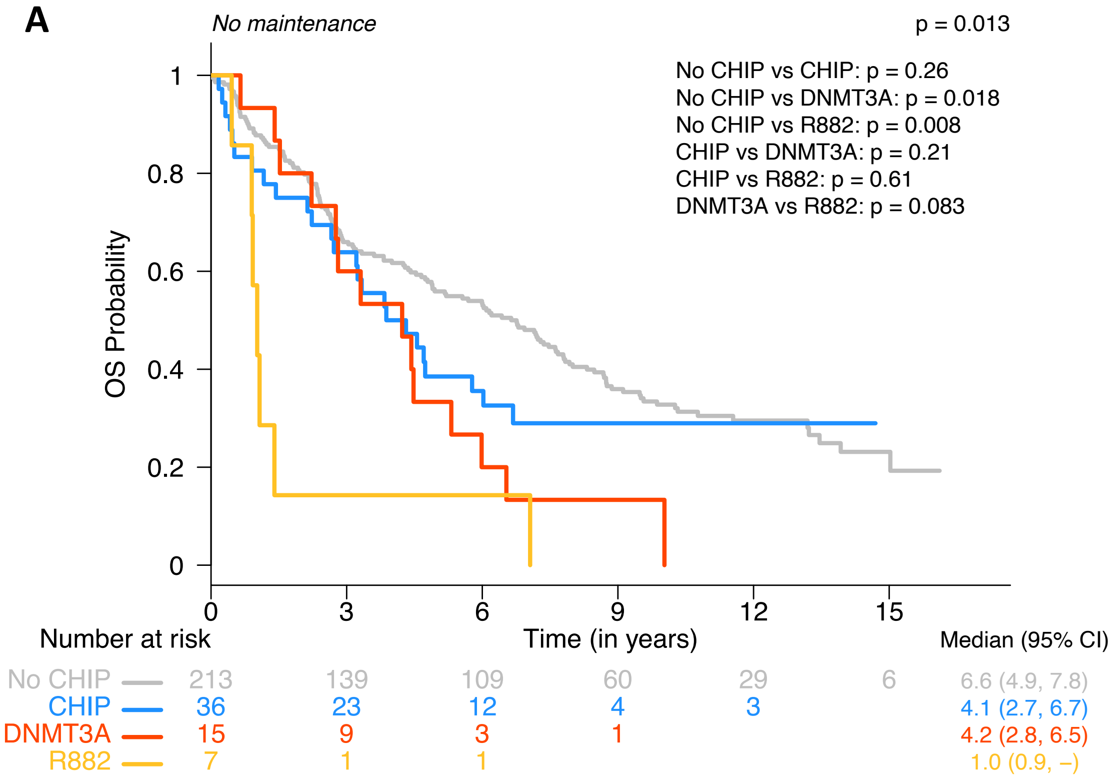

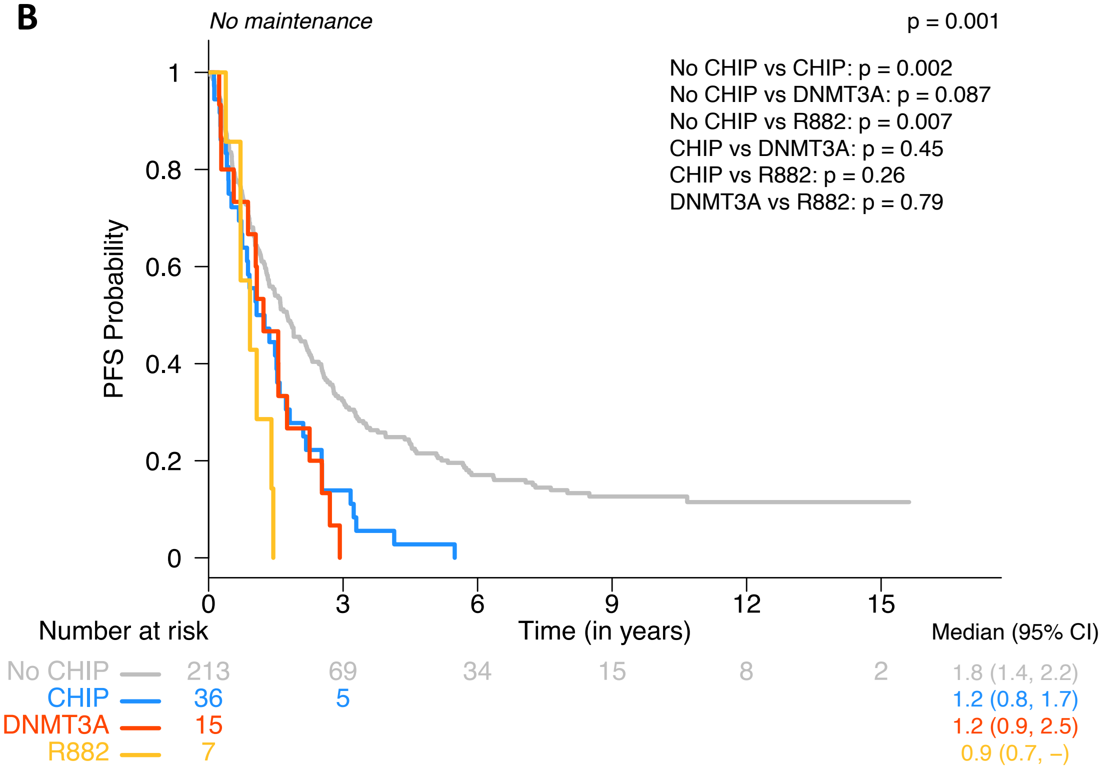

Supplement: Supplementary file 1 — Supplementary Information [file 41467_2020_16805_MOESM1_ESM.pdf]
