# Supplementary material for: Clonal hematopoiesis is associated with adverse outcomes in multiple myeloma patients undergoing transplant
Source: Nat Commun. 2020 Jun 12;11:2996. doi: 10.1038/s41467-020-16805-5 (PMC7293239; doi:10.1038/s41467-020-16805-5)
Supplement: Supplementary file 3 — Reporting Summary [file 41467_2020_16805_MOESM3_ESM.pdf]

nature research

Corresponding author(s): Irene M. Ghobrial

Last updated by author(s): Apr 23, 2020

Reporting Summary

Nature Research wishes to improve the reproducibility of the work that we publish. This form provides structure for consistency and transparency in reporting. For further information on Nature Research policies, see Authors & Referees and the Editorial Policy Checklist .

Statistics

For all statistical analyses, confirm that the following items are present in the figure legend, table legend, main text, or Methods section.

n/a	Confirmed	
☐	☒	The exact sample size (*n*) for each experimental group/condition, given as a discrete number and unit of measurement
☒	☐	A statement on whether measurements were taken from distinct samples or whether the same sample was measured repeatedly
☐	☒	The statistical test(s) used AND whether they are one- or two-sided<br>*Only common tests should be described solely by name; describe more complex techniques in the Methods section.*
☐	☒	A description of all covariates tested
☐	☒	A description of any assumptions or corrections, such as tests of normality and adjustment for multiple comparisons
☐	☒	A full description of the statistical parameters including central tendency (e.g. means) or other basic estimates (e.g. regression coefficient) AND variation (e.g. standard deviation) or associated estimates of uncertainty (e.g. confidence intervals)
☐	☒	For null hypothesis testing, the test statistic (e.g. *F*, *t*, *r*) with confidence intervals, effect sizes, degrees of freedom and *P* value noted<br>*Give P values as exact values whenever suitable.*
☒	☐	For Bayesian analysis, information on the choice of priors and Markov chain Monte Carlo settings
☒	☐	For hierarchical and complex designs, identification of the appropriate level for tests and full reporting of outcomes
☐	☒	Estimates of effect sizes (e.g. Cohen's *d*, Pearson's *r*), indicating how they were calculated

Our web collection on statistics for biologists contains articles on many of the points above.

Software and code

Policy information about availability of computer code

Data collection	No software was used.
Data analysis	R version 3.5.0 (R Core Team), MuTect, Strelka, Orientation Bias Filter, MAFPonFilter, RealignmentFilter, ABSOLUTE, GATK MuTect2, PicardTools, Variant Effect Predictor, and Oncotator.

For manuscripts utilizing custom algorithms or software that are central to the research but not yet described in published literature, software must be made available to editors/reviewers. We strongly encourage code deposition in a community repository (e.g. GitHub). See the Nature Research guidelines for submitting code & software for further information.

Data

Policy information about availability of data

All manuscripts must include a data availability statement. This statement should provide the following information, where applicable:

- Accession codes, unique identifiers, or web links for publicly available datasets
- A list of figures that have associated raw data
- A description of any restrictions on data availability

Sequencing data and basic phenotype data can be accessed via dbGaP accession code phs001323. All other remaining data are available within the article and supplementary files, or available from the authors upon request.

Field-specific reporting

Please select the one below that is the best fit for your research. If you are not sure, read the appropriate sections before making your selection.

October 2018

Life sciences study design

All studies must disclose on these points even when the disclosure is negative.

Sample size	We collected the clinical data and all available cryopreserved aliquots of mobilized autologous stem-cell products from 629 MM patients who underwent ASCT between January 2003 and December 2011 at the Dana-Farber Cancer Institute in Boston, MA.
Data exclusions	No data was excluded from the analyses.
Replication	The findings of this study can be replicated/reproduced if another large cohort of multiple myeloma patients is available for sequencing and clinical correlation.
Randomization	This study is a retrospective study and randomization is not possible.
Blinding	Investigators were blinded to group allocation (CHIP vs no CHIP) while collecting the clinical data.

Reporting for specific materials, systems and methods

We require information from authors about some types of materials, experimental systems and methods used in many studies. Here, indicate whether each material, system or method listed is relevant to your study. If you are not sure if a list item applies to your research, read the appropriate section before selecting a response.

Materials & experimental systems

n/a	Involved in the study	
☒	☐	Antibodies
☒	☐	Eukaryotic cell lines
☒	☐	Palaeontology
☒	☐	Animals and other organisms
☐	☒	Human research participants
☒	☐	Clinical data

Methods

n/a	Involved in the study	
☒	☐	ChIP-seq
☒	☐	Flow cytometry
☒	☐	MRI-based neuroimaging

Human research participants

Policy information about studies involving human research participants

Population characteristics	Female and male multiple myeloma patients above the age of 18.
Recruitment	We do not perceive the presence of any selection bias since we included all myeloma patients who presented to Dana-Farber Cancer Institute and were treated and received an autologous stem cell transplant between 2003 and 2011.
Ethics oversight	The Dana-Farber Cancer Institute IRB.

Note that full information on the approval of the study protocol must also be provided in the manuscript.
